# Cell shape sensing licenses dendritic cells for homeostatic migration to lymph nodes

**DOI:** 10.1038/s41590-024-01856-3

**Published:** 2024-06-04

**Authors:** Zahraa Alraies, Claudia A. Rivera, Maria-Graciela Delgado, Doriane Sanséau, Mathieu Maurin, Roberto Amadio, Giulia Maria Piperno, Garett Dunsmore, Aline Yatim, Livia Lacerda Mariano, Anna Kniazeva, Vincent Calmettes, Pablo J. Sáez, Alice Williart, Henri Popard, Matthieu Gratia, Olivier Lamiable, Aurélie Moreau, Zoé Fusilier, Lou Crestey, Benoit Albaud, Patricia Legoix, Anne S. Dejean, Anne-Louise Le Dorze, Hideki Nakano, Donald N. Cook, Toby Lawrence, Nicolas Manel, Federica Benvenuti, Florent Ginhoux, Hélène D. Moreau, Guilherme P. F. Nader, Matthieu Piel, Ana-Maria Lennon-Duménil

**Affiliations:** 1 INSERM U932, Immunity and Cancer, Institut Curie, PSL University, Paris, France; 2 Cellular Immunology, International Centre for Genetic Engineering and Biotechnology (ICGEB), Trieste, Italy; 3 INSERM U1015, Gustave Roussy Cancer Campus, Villejuif, France; 4 Cell Communication and Migration Laboratory, Institute of Biochemistry and Molecular Cell Biology, Center for Experimental Medicine, University Medical Center Hamburg-Eppendorf, Hamburg, Germany; 5 CNRS UMR144, Institut Curie, PSL Research University, Paris, France; 6 Malaghan Institute of Medical Research, Wellington, New Zealand; 7 Center for Research in Transplantation and Translational Immunology, UMR 1064, INSERM, Nantes Université, Nantes, France; 8 INSERM U932, Immunity and Cancer, Institut Curie, Paris-Cité University, Paris, France; 9 Platform NGS-ICGEX, Institut Curie, Paris, France; 10 INSERM UMR1291, CNRS UMR5051, Institut Toulousain des Maladies Infectieuses et Inflammatoires (INFINITy), Université Toulouse III, Toulouse, France; 11 Immunity, Inflammation, and Disease Laboratory, National Institute of Environmental Health Sciences (NIEHS), National Institutes of Health (NIH), Research Triangle Park, NC USA; 12 Centre d’Immunologie de Marseille-Luminy, INSERM, CNRS, Université Aix-Marseille, Marseille, France; 13 Centre for Inflammation Biology and Cancer Immunology, School of Immunology and Microbial Sciences, King’s College London, London, UK; 14 Henan Key Laboratory of Immunology and Targeted Therapy, School of Laboratory Medicine, Xinxiang Medical University, Xinxiang, China; 15 Singapore Immunology Network (SIgN), Agency for Science, Technology and Research (A*STAR), 8A Biomedical Grove, Immunos, Singapore, Singapore; 16 Department of Immunology and Microbiology, Shanghai Institute of Immunology, Shanghai Jiao Tong University School of Medicine, Shanghai, China; 17 Translational Immunology Institute, SingHealth Duke-NUS Academic Medical Centre, Singapore, Singapore; 18 Department of Pathology and Laboratory Medicine, Children’s Hospital of Philadelphia and University of Pennsylvania Perelman School of Medicine, Philadelphia, PA USA

**Keywords:** Conventional dendritic cells, Chemotaxis, Chemokines

## Abstract

Immune cells experience large cell shape changes during environmental patrolling because of the physical constraints that they encounter while migrating through tissues. These cells can adapt to such deformation events using dedicated shape-sensing pathways. However, how shape sensing affects immune cell function is mostly unknown. Here, we identify a shape-sensing mechanism that increases the expression of the chemokine receptor CCR7 and guides dendritic cell migration from peripheral tissues to lymph nodes at steady state. This mechanism relies on the lipid metabolism enzyme cPLA_2_, requires nuclear envelope tensioning and is finely tuned by the ARP2/3 actin nucleation complex. We also show that this shape-sensing axis reprograms dendritic cell transcription by activating an IKKβ–NF-κB-dependent pathway known to control their tolerogenic potential. These results indicate that cell shape changes experienced by immune cells can define their migratory behavior and immunoregulatory properties and reveal a contribution of the physical properties of tissues to adaptive immunity.

## Abstract (web summary)

Dendritic cells experience cell shape changes while migrating within the complex physical environment of tissues. Sensing of these shape changes modifies their migratory properties and imprints these cells with immunoregulatory properties.

## Main

The success of immune responses largely relies on the motility of immune cells that constantly circulate between peripheral tissues and/or lymphoid organs. To migrate, these cells must exert forces on the environment, which in most cases is achieved through the dynamic reorganization of their actomyosin cytoskeleton in response to external signals^1^. In addition, to reach a particular destination, cells sense biochemical and/or physical cues that guide them through the complex environment of tissues and vessels^2^. Among them, chemokines, which can form gradients recognized by specific cell-surface receptors, are known to play a prominent role^3^. It is well known that various types of biochemical signals can switch on the expression of these receptors. However, whether chemokine receptor expression responds to the physical stimuli encountered by migrating immune cells is unclear. A chemokine receptor that has attracted a lot of attention is CCR7, which recognizes gradients of CCL19 and CCL21 chemokines^4,5^. CCR7 is required for the migration of distinct types of immune cells to lymph nodes, including dendritic cells (DCs). Once in lymph nodes, DCs present antigens collected in their tissue of residency to T cells^6,7^. Antigen presentation has two distinct outcomes^8^. It leads to T cell activation when DCs present antigens from a tissue that is inflamed because of infection or tumor growth. Alternatively, it can inactivate self-reactive T cells, a process referred to as ‘peripheral immune tolerance’, which limits autoimmune reactions at steady state^7,9,10^. Interestingly, a population of CCR7^+^ DCs was recently described as residing within tumors^11,12^; it remains unclear whether these cells trigger tolerogenic or antitumoral responses. Regulation of CCR7 expression might thus be critical to define the balance between tolerance and immunity at steady state or during infection and cancer. The main inducers of CCR7 expression identified so far are microbial components and endogenous inflammatory mediators^4,13,14^. Interestingly, seminal studies from the Mellman group have shown that mechanical disruption of cell–cell junctions can also induce the expression of CCR7 in DCs^15^. However, whether this phenomenon contributes to DC migration to lymph nodes remains an open question, as it is unclear whether DCs form such junctions within peripheral tissues. Nonetheless, these results suggest that the physical signals to which DCs are exposed while patrolling peripheral tissues might modify the capacity of DCs to express CCR7 and migrate to lymph nodes in the absence of tissue inflammation^15–17^.

In tissues, a major physical signal experienced by motile cells results from the shape changes and deformation of internal organelles imposed by the physical constraints that they experience. We and others have shown that immune cells can adapt and respond to large shape changes when spontaneously migrating through peripheral tissues or dense tumors^18–20^. Such shape changes lead to nuclear deformation events that can activate the lipid metabolism enzyme cytosolic phospholipase 2 (cPLA_2_), a sensor of nuclear envelope stretching^21–23^. Once activated, cPLA_2_ uses phospholipids from the nuclear membrane to produce arachidonic acid (AA) that can be converted into leukotriens and prostaglandins^24,25^. AA production by cPLA_2_ also enhances actomyosin contractility, allowing cells that are physically trapped within a dense tissue to release themselves and keep moving forward^21,23^. Whether cPLA_2_ activation has any impact on the transcriptional activity of immune cells is unknown.

Here, we subjected DCs to distinct deformation events of amplitudes that fall within the range of shape changes that they experience in vivo. We identified a precise deformation amplitude at which DCs turn on an ARP2/3–cPLA_2_–NF-κB-dependent shape-sensing mechanism. Activation of this pathway leads to CCR7 upregulation, controls steady-state DC migration to lymph nodes and further reprograms transcription in a different way than microbial sensing. Hence, DCs use the cytoskeleton–lipid metabolism interplay to respond to environmental physical constraints that imprint them with specific immunoregulatory properties.

## Results

### DC motility and CCR7 expression are co-regulated after shape changes

**Fig. 1:**
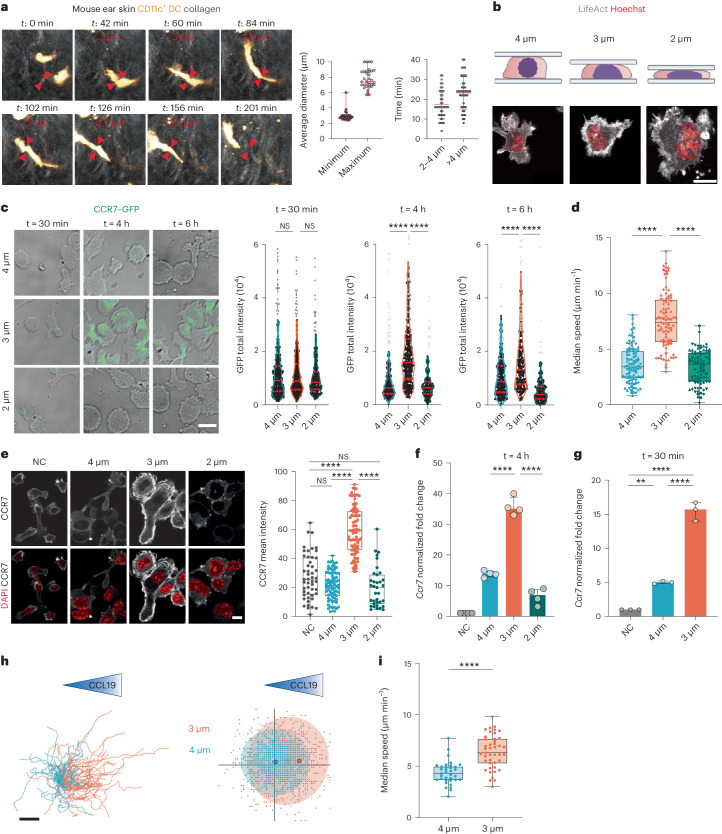
CCR7 upregulation in immature DCs is shape sensitive. **a**, Left, migrating CD11c^+^ DCs in an ear explant. Arrows highlight cell deformation events. Center, minimum to maximum range of median cell diameter (*n* = 34 cells). Right, average time spent by each cell at their minimum diameter (mean ± s.d.; *n* = 43 cells). **b**, Top, schematic of cells under confinement (created with BioRender.com). Bottom, representative images of live DCs expressing LifeAct–GFP (gray) and DNA (red); scale bar, 10 µm. **c**, Left, representative images of CCR7–GFP-expressing DCs; scale bar, 10 µm. Right, violin plot (median and quartiles) of total GFP intensity. Outliers were calculated using a ROUT test (*Q* = 1%) represented in gray (*N* = 4 independent experiments; left, *n* = 348 cells in 4 µm, *n* = 314 cells in 3 µm, *n* = 211 cells in 2 µm; middle, *n* = 201 cells in 4 µm, *n* = 206 cells in 3 µm, *n* = 176 cells in 2 µm; right, *n* = 215 cells in 4 µm, *n* = 187 cells in 3 µm, *n* = 180 cells in 2 µm). Data were analyzed by Kruskal–Wallis test; ****P < 0.0001; NS, not significant (*P* > 0.999). **d**, Box plot with minimum to maximum range of median speed (*N* = 4 independent experiments; *n* = 96 cells in 4 µm, *n* = 89 cells in 3 µm, *n* = 85 cells in 2 µm). Data were analyzed by Kruskal–Wallis test; ****P < 0.0001. **e**, Left, representative images of DCs showing CCR7 expression (gray) and nuclei (red). A maximum *z* projection is shown; scale bar, 10 µm. Right, box plot with minimum to maximum range (*N* = 2 independent experiments; *n* = 49 nonconfined cells, *n* = 74 cells in 4 µm, *n* = 89 cells in 3 µm, *n* = 35 cells in 2 µm). Data were analyzed by Kruskal–Wallis test; ****P < 0.0001; NS, *P* > 0.999; NC, nonconfined. **f**, Expression of *Ccr7*. Data are shown as mean ± s.d. (*N* = 4 independent experiments). Data were analyzed by Kruskal–Wallis test; ****P < 0.0001. **g**, Expression of *Ccr7* after 30 min of confinement. Data are shown as mean ± s.d. (*N* = 3 independent experiments). Data were analyzed by Kruskal–Wallis test; **P = 0.0021; ****P < 0.0001. **h**, Left, cell trajectories; scale bar, 100 µm. Middle, representation of cell step. Data are representative of two independent experiments (*n* = 33 cells in 4 µm; *n* = 43 cells in 3 µm). **i**, Box plot with minimum to maximum range of mean speed. Data were analyzed by one-way Mann–Whitney test; ****P < 0,0001. Data are representative of two independent experiments (*n* = 33 cells in 4 µm; *n* = 43 cells in 3 µm). Source data

**Extended Data Fig. 1:**
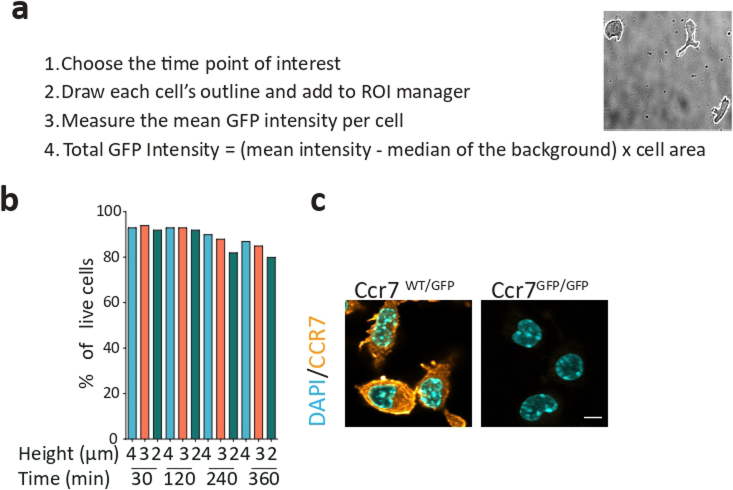
GFP quantification approach. (**a**) Quantification approach to quantify GFP intensity in each cells. (**b**) Quantification of cell viability in different confinement conditions and during different time points, using propidium iodide (**c**) representative images of immature DCs confined for 4 h at the height of 3 µm from C57BL/6 J (WT) or GFP/GFP mice (*Ccr7*KO), CCR7 was visualized using immunostaining (in orange) and the nucleus stained with DAPI (in cyan) scale bar 5 µm. Source data

To migrate to lymph nodes, DCs must express the CCR7 chemokine receptor and enhance their intrinsic motility. We have previously observed that confinement-induced stretching of the nuclear envelope increases DC motility by promoting actomyosin contractility in a cPLA_2_-dependent manner^21^. We hypothesized that such shape change might further trigger CCR7 expression, thereby endowing DCs with the full capacity to reach their next destination. To test this hypothesis, we manipulated the shape of DCs in a controlled manner using a cell-confining device^26^. To define the confinement heights to be used, we imaged DCs patrolling the dermis of mouse ear explants by two-photon microscopy. Timelapse movies showed that DCs expressing yellow fluorescent protein-tagged CD11c (CD11c–YFP) exhibited a minimal cell diameter of, on average, ∼2–4 µm and a maximal cell diameter of ∼6–10 µm while sampling the skin (Fig. 1a and Supplementary Video 1). We further observed that DCs spent ∼35% of their time displaying diameters between ∼2 and 4 µm and spent ∼50% of their time at diameters of >4 µm (Fig. 1a). These results were in good agreement with data previously obtained by measuring the minimal diameters of the nuclei of bone marrow-derived DCs transferred into epidermal ear sheets^19^. This prompted us choosing confinement heights of 2, 3 and 4 μm to monitor CCR7 expression in DCs experiencing cell shape changes (Fig. 1b). As a cell model, we used unstimulated bone marrow-derived DCs (immature DCs), which express low levels of CCR7 in culture^27,28^, similar to DCs patrolling peripheral tissues^29^. Of note, tissue-resident DCs could not be used for our purpose as they spontaneously upregulate CCR7 expression after tissue disruption. Bone marrow was obtained from a mouse model where a reporter gene encoding green fluorescent protein (GFP)-tagged CCR7 (CCR7–GFP) was knocked in to evaluate the dynamics of CCR7 expression in deformed DCs^30^. Strikingly, fluorescence quantification showed that although GFP expression increased in DCs confined at a height of 3 µm for 4–6 h (Extended Data Fig. 1a), it was not substantially modified in cells confined at heights of 2 or 4 µm (Fig. 1c and Supplementary Video 2). GFP upregulation did not result from cell death, which was low at all confinement heights (<8% of dead cells; Extended Data Fig. 1b). A similar sensitivity window was observed when monitoring the migration speed of confined DCs, which increased at a confinement height of 3 µm but not at heights of 2 or 4 µm (Fig. 1d). These experiments indicate that both CCR7 expression and cell motility respond to precise cell shape changes, suggesting that these events might be mechanistically coupled.

We confirmed these results when analyzing endogenous CCR7 expression by real-time quantitative PCR with reverse transcription (RT–qPCR) and immunofluorescence. Only immature DCs confined at a height of 3 μm exhibited a significant increase in CCR7 expression at both mRNA and protein levels (Fig. 1e,f). The specificity of the antibody to CCR7 was verified using *Ccr7*-knockout DCs (Extended Data Fig. 1c). *Ccr7* mRNA expression was also induced when cells were confined for 30 min, collected and analyzed 4 h later (Fig. 1g), suggesting that a 30-min deformation at a height of 3 μm was sufficient to upregulate *Ccr7* expression in DCs. Of note, CCR7 expressed at the surface of DCs confined at a height of 3 µm was fully functional; only these cells exhibited chemotaxis when CCL19 was added at one side of the confinement device (Fig. 1h,i). Together, these results show that confinement of immature DCs at a height of 3 μm, but not at 2 or 4 μm, upregulates CCR7 expression and endows these cells with the ability to perform chemotaxis and enhances their intrinsic motility. DC confinement at a height of 4 μm was thus chosen as the negative-control condition for all following experiments.

### CCR7 upregulation after shape sensing relies on cPLA_2_ activity

**Fig. 2:**
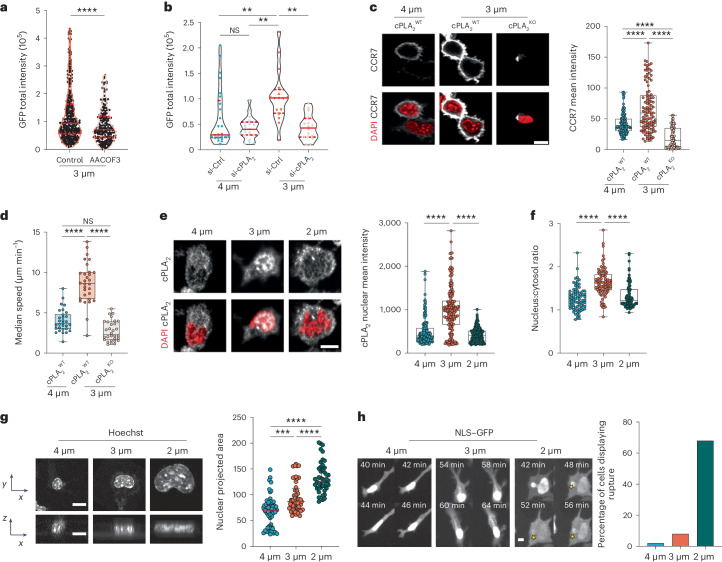
CCR7 upregulation in response to shape sensing depends on cPLA_2_ and an intact nuclear envelope. **a**, GFP intensity in DCs treated with the cPLA_2_ inhibitor AACOF3 (25 µM) or control. The violin plot shows median values and quartiles (*N* = 3, *n* = 348 cells in the 3-µm control, *n* = 225 cells treated with AACOF3). Data were analyzed by one-way Mann–Whitney test; ****P < 0.0001. **b**, Expression of CCR7–GFP in *Pla2g4a*-knockdown (si-cPLA_2_) and control (si-Ctrl) DCs. The violin plot shows median values and quartiles (*N* = 2, *n* = 25 cells in si-Ctrl at 4 µm (80% of cells), *n* = 21 cells in si-cPLA_2_ at 4 µm (75% of cells), *n* = 16 cells in si-Ctrl at 3 µm (75% of cells), *n* = 16 cells in si-cPLA_2_ at 3 µm (76% of cells)). Data were analyzed by one-way analysis of variance (ANOVA) with a Kruskal–Wallis multiple comparison analysis; **P = 0.0059; **P = 0.0039. **c**, Left, representative images of cPLA_2_^WT^ and cPLA_2_^KO^ DCs. CCR7 is in gray, and nuclei are in red; scale bar, 10 µm. Right, box plot with minimum to maximum range of CCR7 intensity (*N* = 3, *n* = 114 cells in cPLA_2_^WT^ at 4 µm, *n* = 105 cells in cPLA_2_^WT^ at 3 µm, *n* = 86 cells in cPLA_2_^KO^ at 3 µm). Data were analyzed by Kruskal–Wallis test; ****P < 0.0001. **d**, Box plot showing the minimum to maximum range of median speed of cPLA_2_^WT^ and cPLA_2_^KO^ DCs (*N* = 2, *n* = 30 cells in all positions). Data were analyzed by Kruskal–Wallis test; ****P < 0.0001; NS, *P* > 0.999. **e**, Left, representative images of confined DCs. cPLA_2_ is in gray, and nuclei are in red; scale bar, 10 µm. Right, box plot showing the minimum to maximum range of cPLA_2_ intensity (*N* = 3, *n* = 165 cells in 4 µm, *n* = 178 cells in 3 µm, *n* = 230 cells in 2 µm). Data were analyzed by Kruskal–Wallis test; ****P < 0.0001. **f**, Nucleus-to-cytosolic ratio of cPLA_2_ expression. The box plot shows the minimum to maximum range (*N* = 3, *n* = 165 cells in 4 µm, *n* = 178 cells in 3 µm, *n* = 230 cells in 2 µm). Data were analyzed by Kruskal–Wallis test; ****P < 0.0001. **g**, Left, projection of cell nuclei; scale bar, 10 µm. Right, box plot showing the median and interquartile range of the nucleus area (*N* = 3, *n* = 55 cells in 4 µm, *n* = 49 cells in 3 µm, *n* = 44 cells in 2 µm). Data were analyzed by Kruskal–Wallis test; ****P < 0.0001; ***P < 0.0006. **h**, Left, images of DCs transduced with NLS–GFP. Yellow stars indicate nuclear envelope rupture. Right, percentage of DCs displaying rupture events (*n* = median of 3 independent experiments). Source data

**Extended Data Fig. 2:**
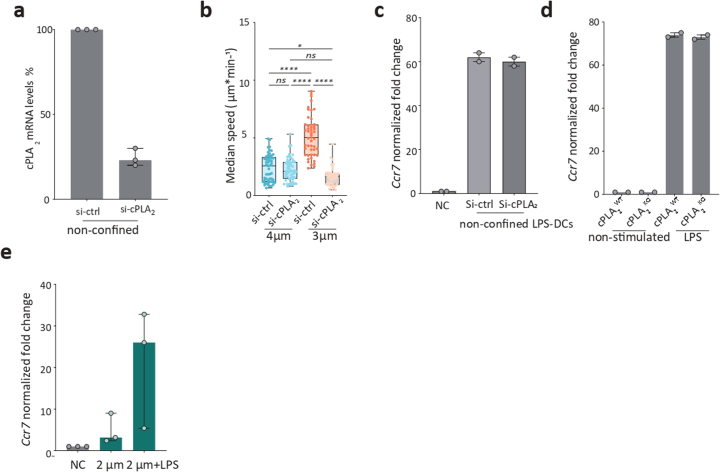
cPLA_2_ activity is not necessary for LPS-induced *Ccr7* up regulation. (**a**) median with interquartile range of cPLA_2_^gene expression upon its knock down, N^ = ^3, n^ = ^9^ (**b**) Box plot with min to max range of median speed of cPLA_2_ KD or control DCs. N = 3, n = 53 cells in cPLA_2_ Ctrl4µm, n = 53 cells in cPLA_2_ KD 4µm, n = 49 cells in cPLA_2_ Ctrl 3 µm, n = 44 cells in cPLA_2_ KD 3 µm. one way ANOVA with Kruskal-Wallis multiple analysis test, ****:P < 0.0001, *: P = 0.0315, ns: not significant P = 0.0813. (**c**) RT-qPCR data of *Ccr7* gene expression shows no difference in *Ccr7* expression in cPLA_2_ KD or control cells activated with the microbial component LPS, median with interquartile range of 2 independent experiments, n = 6 (**d**) RT-qPCR data of *Ccr7* gene expression in cPLA_2_ WT and KO DCs activated with LPS ^activated with LPS^. median with interquartile range of 3 independent experiments. (**e**) RT- qPCR data of *Ccr7* gene expression in cells confined at 2 µm activated with LPS after confinement, median with interquartile range of 3 independent experiments, n = 9. Source data

We found that both CCR7 expression and DC motility were induced at the same confinement height (3 μm), suggesting that these processes might be controlled by common mechanisms. We thus investigated whether CCR7 upregulation involves cPLA_2_, as we had previously shown that this lipid metabolism enzyme enhances cell motility in response to nuclear deformation^21,23^. Inhibiting the enzymatic activity of cPLA_2_ by treatment with the drug AACOCF3 prevented the upregulation of CCR7–GFP expression in DCs confined at a height of 3 µm (Fig. 2a). Similar results were observed in *Pla2g4a-*knockdown DCs, which further exhibited decreased cell motility (Fig. 2b and Extended Data Fig. 2a,b). To strengthen these results, we generated conditional knockout mice for the *Pla2g4a* gene (*Pla2g4a*^fl/fl^ crossed with *Itgax-cre* (Cd11c-cre) transgenic animals to obtain *Pla2g4a*-knockout (cPLA_2_^KO^) DCs). These cells did not upregulate CCR7 expression nor increase their motility after confinement at a height of 3 µm (Fig. 2c,d). By contrast, *Pla2g4a* knockdown or knockout did not prevent *Ccr7* upregulation in response to treatment with the microbial compound lipopolysaccharide (LPS; Extended Data Fig. 2c,d). These results indicate that cPLA_2_ is specifically required for CCR7 expression induced by cell shape changes rather than being generally involved in the transcriptional regulation of the *Ccr7* gene. We conclude that upregulation of both cell motility and CCR7 expression in DCs confined at a height of 3 µm relies on the activity of the cPLA_2_ enzyme.

Consistently, we observed that cPLA_2_ accumulated in the nucleus of DCs confined at a height of 3 µm, but not at 2 or 4 µm (Fig. 2e,f). cPLA_2_ is known to translocate to the nucleus^31^ and associate with the inner nuclear membrane after activation^22^. Analysis of nuclear shape showed that DCs confined at heights of both 3 and 2 μm gradually increased the nucleus projected area (Fig. 2g), consistent with the nuclei being more deformed than the nuclei of cells confined at 4 µm. These results show that cPLA_2_ does not accumulate in the nuclei of DCs confined at a height of 2 µm despite their nucleus being extensively stretched. This finding prompted us to hypothesize that confinement at a height of 2 µm might compromise nuclear accumulation of cPLA_2_ due to loss of nuclear envelope integrity. To test this hypothesis, we transduced DCs with a lentiviral construct expressing nuclear localization signal (NLS)-tagged GFP (NLS–GFP). We observed that most NLS–GFP-expressing DCs confined at a height of 2 µm underwent nuclear envelope rupture followed by repair events, as evidenced by the transient leakage of NLS–GFP signal into the cytoplasm (Fig. 2h and Supplementary Video 3). Nuclear envelope rupture was less frequently observed in DCs confined at a height of 3 or 4 µm (Fig. 2h). Of note, DCs confined at a height of 2 µm did not display any additional sign of damage and were able to upregulate *Ccr7* expression after treatment with microbial LPS (Extended Data Fig. 2e). Together, these results show that coordinated upregulation of DC motility and CCR7 expression relies on nuclear accumulation of cPLA_2_, which requires an intact nuclear envelope, and point to DCs being equipped with an extremely accurate machinery to detect precise levels of cell shape changes.

### ARP2/3 activity defines the cPLA_2_ activation threshold after shape sensing

**Fig. 3:**
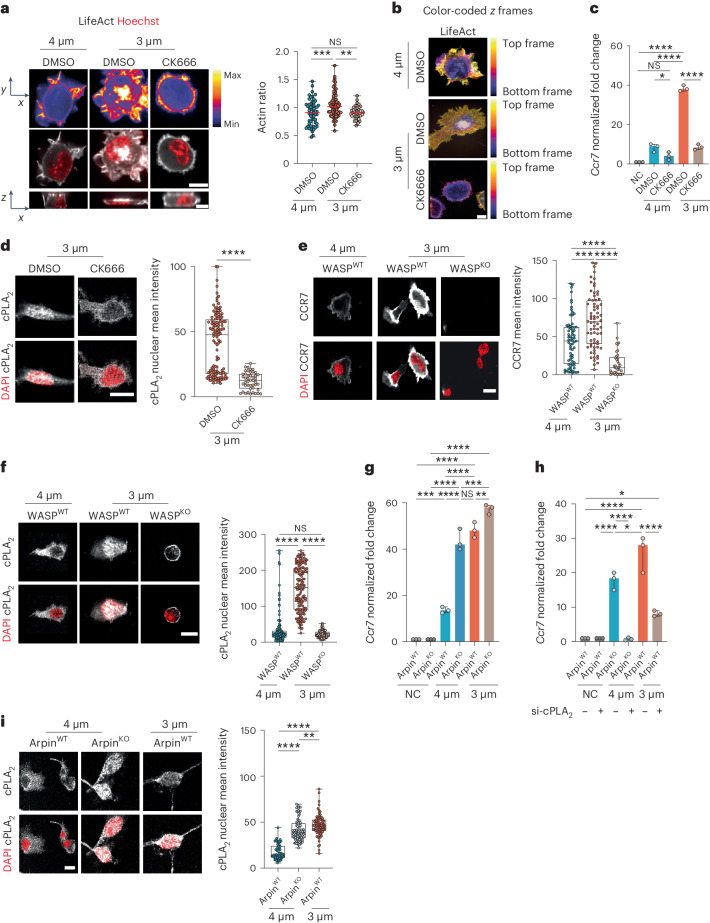
ARP2/3 activity tunes the sensitivity of the cPLA2-dependent shape-sensing DC response. **a**, Top, representative images of LifeAct–GFP DCs treated with CK666 (30 µM) or untreated. Middle, images of LifeAct–GFP (gray) and nuclei (red); scale bar 10 µm. Bottom, single resliced image; scale bar, 20 µm. Right, median with range of LifeAct nucleus-to-cytosol ratio (*N* = 3; *n* = 55 cells in 4 µm (DMSO), *n* = 70 cells in 3 µm (DMSO), *n* = 42 cells in 3 µm (CK666)). Data were analyzed by ordinary one-way ANOVA; ***P = 0.0007; **P = 0.0016. **b**, Color-coded *z* frames of untreated LifeAct DCs and cells treated with 30 µM CK666. **c**, Expression of *Ccr7* (mean ± s.d.; *N* = 3). Data were analyzed by ordinary one-way ANOVA; ****P < 0.0001; *P = 0.0465; NS, *P* > 0.999. **d**, Left, representative images of DCs treated with 30 µM CK666 or untreated cells. cPLA_2_ is in gray, and nuclei are in red; scale bar, 10 µm. Right, box plot showing the minimum to maximum range of cPLA_2_ intensity (*N* = 2; *n* = 121 cells in DMSO (3 µm), *n* = 64 cells in CK666 (3 µm)). Data were analyzed by one-way Mann–Whitney test; ****P < 0.0001. **e**, Left, representative images of WASp^WT^/WASp^KO^ DCs. CCR7 is in gray, and nuclei are in red; scale bar, 10 µm. Right, box plot showing the minimum to maximum range of CCR7 intensity (*N* = 2; *n* = 70 cells in WASp^WT^ at 4 µm, *n* = 70 cells in WASp^WT^ at 3 µm, *n* = 38 cells in WASp^KO^ at 3 µm). Data were analyzed by Kruskal–Wallis test; ****P < 0.0001; ***P < 0.0002. **f**, Left, representative images of WASp^WT^/WASp^KO^ DCs. cPLA_2_ is in gray, and the nuclei are in red; scale bar, 10 µm. Right, box plot showing the minimum to maximum range of cPLA_2_ intensity (*N* = 3; *n* = 150 cells in WASp^WT^ at 4 µm, *n* = 145 cells in WASp^WT^ at 3 µm, *n* = 149 cells in WASp^KO^ at 3 µm). Data were analyzed by Kruskal–Wallis test; ****P < 0.0001. **g**, Expression of *Ccr7* in Arpin^WT^/Arpin^KO^ DCs. Data are shown as mean ± s.d. (*N* = 3) and were analyzed by ordinary one-way ANOVA; ****P < 0.0001; ***P = 0.0002; **P = 0.0015; NS, *P* > 0.999. **h**, Expression of *Ccr7* in Arpin^WT^/Arpin^KO^ DCs (*N* = 3). Data were analyzed by ordinary one-way ANOVA; ****P < 0.0001; *P = 0.0134. **i**, Left, representative images of Arpin^WT^/Arpin^KO^ DCs. cPLA_2_ is in gray, and nuclei are in red; scale bar, 10 µm. Right, box plot showing the minimum to maximum range of cPLA_2_ intensity (*N* = 2; *n* = 59 cells in Arpin^WT^ at 4 µm, *n* = 87 cells in Arpin^KO^ at 4 µm, *n* = 75 cells in Arpin^WT^ at 3 µm). Data were analyzed by ordinary one-way ANOVA; ****P < 0.0001; **P = 0.0043. Source data

**Extended Data Fig. 3:**
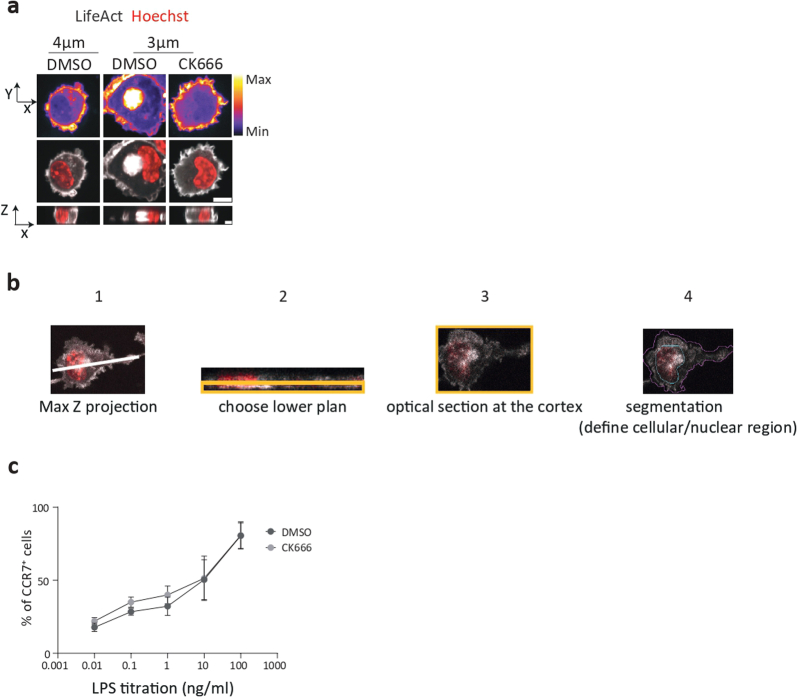
Arp2/3 branched actin is not important for CCR7 upregulation upon LPS. (**a**) Second example: Upper panel: representative XY images of DCs expressing LifeAct-GFP (false colors), Middle panel: XY images of LifeAct-GFP DCs (in grey) stained with NucBlue (DNA, red) treated or not with CK666 (30 µM), scale bar 10 µ. lower panel: XZ view, single confocal frame from resliced images, scale bar 20 µ. (**b**) Quantification approach of LifeAct-GFP intensity in cells under confinement: 1- choose Z-stack lower plan (since cells don’t always touch the upper plan). 2^−^ optical section at the surface cortex. 3-Segmentation to define cell and nuclear contour at the surface. 4- Measurement of LifeAct-GFP ratio: Actin Ratio= Nuclear surface mean actin intensity/cell surface mean actin intensity (ratio <1 => actin is mostly cortical). (**c**) FACS analysis of CCR7^+^ DCs activated by different concentration of LPS and treated with CK666 (30 µM) or DMSO. N = 3 independent experiments. Source data

We next asked whether nuclear translocation and activation of cPLA_2_ resulted from passive stretching of the DC nucleus after confinement or instead required an active cellular response. A good candidate for driving such a response was ARP2/3, as this complex has been shown to nucleate distinct types of actin structures in the perinuclear area of DCs undergoing nucleus deformation^18,32^. Live imaging analysis of LifeAct–GFP distribution in DCs as soon as they were confined at a height of 3 µm indeed revealed a cloud of perinuclear F-actin present in ∼45% of cells compared to ∼10% of cells under control conditions (Fig. 3a). Of note, this actin cloud was only observed when cells were fixed under confinement and was positioned on top or on either side of the nucleus (Fig. 3a and Extended Data Fig. 3a,b). Treatment of DCs with CK666, which inhibits ARP2/3 activity, led to the disappearance of this actin structure (Fig. 3a, Supplementary Video 4 and Extended Data Fig. 3a), suggesting that ARP2/3 activity is needed for its formation. This result was confirmed using a color-coded *z* projection to analyze imaged cells. In DCs confined at a height of 3 μm, most actin was found in a cloud located on the top of nuclei (Fig. 3b).

We found that CK666 impaired the upregulation of *Ccr7* expression and the nuclear accumulation of cPLA_2_ after confinement at a height of 3 µm (Fig. 3c,d). As observed for cPLA_2_, ARP2/3 inhibition had no effect on LPS-induced CCR7 upregulation (Extended Data Fig. 3c). Consistent with these results, we further found that DCs lacking Wiscott–Aldrich syndrome protein (WASp; encoded by the gene *Was*), which was recently shown to activate ARP2/3 in the DC perinuclear area^33^, behaved similar to cells treated with CK666; they did not upregulate CCR7 expression nor show cPLA_2_ nuclear translocation after confinement at a height of 3 µm (Fig. 3e,f). Of note, *Was*-knockout (WASp^KO^) DCs exhibited a round morphology, consistent with previous reports by others^34^ (Supplementary Video 5). Together, these results suggest that the response of DCs to shape changes requires WASp and ARP2/3 activity to allow nuclear accumulation of cPLA_2_ and subsequent upregulation of CCR7 expression.

To strengthen these findings, we assessed the response of DCs lacking the ARP2/3 inhibitor Arpin (*Arpin*^fl/fl^ × *Itgax*-*cre*), which exhibit enhanced ARP2/3 activity^35^. Remarkably, we found that *Arpin*-knockout (Arpin^KO^) immature DCs displayed an increased sensitivity to cell shape changes as they upregulated *Ccr7* when confined at a height of 4 µm instead of 3 µm (Fig. 3g) in a cPLA_2_-dependent manner (Fig. 3h). Accordingly, Arpin^KO^ cells confined at 4 µm also exhibited cPLA_2_ nuclear translocation (Fig. 3i). We conclude that the activity of ARP2/3 determines the sensitivity of DCs to confinement, defining a threshold for cPLA_2_ nuclear accumulation and induction of CCR7 expression in response to cell shape changes. These results are consistent with DCs being equipped with a sensory mechanism that finely tunes their response to shape changes.

### ARP2/3 triggers cPLA_2_ activation via nuclear envelope tensioning

**Fig. 4:**
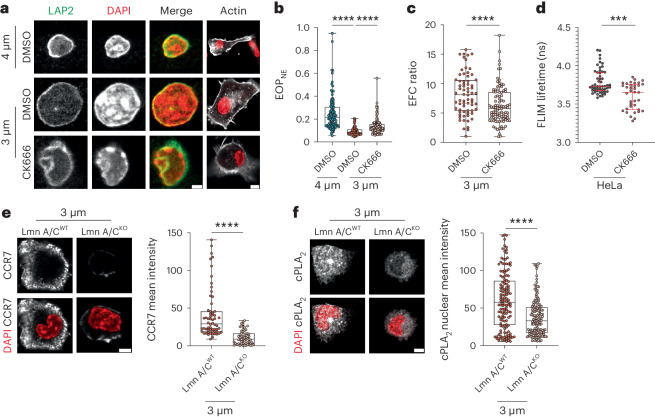
ARP2/3 activity mediates DC shape sensing by unfolding the nuclear envelope and increasing its tension. **a**, Representative images of DCs treated with 30 µM CK666 or untreated. LAP2 and DAPI are in gray on the left. Merged images of DAPI (red) and LAP2 (green) and of DAPI (red) and phalloidin (gray) are shown on the right; scale bars, 2 µm (left merge) and 10 µm (right merge). Images are representative of *N* = 2 samples. **b**, EOP_NE_ of confined DCs treated with 30 µM CK666 or untreated DCs. Box plots show the minimum to maximum range (*N* = 3; *n* = 100 cells in 4 µm (DMSO), *n* = 93 cells in 3 µm (DMSO), *n* = 97 cells in 3 µm (CK666)). Data were analyzed by ordinary one-way ANOVA; ****P < 0.0001. **c**, EFC of confined DCs treated with 30 µM CK666 or untreated DCs. Box plots show the minimum to maximum range (*N* = 3; *n* = 68 cells in 3 µm (DMSO), *n* = 97 cells in 3 µm (CK666)). Data were analyzed by one-way Mann–Whitney test; ****P < 0.0001. **d**, Nuclear envelope tension sensor FLIM measurements in HeLa cells after treatment with 30 µM CK666. Data are shown as median values with the interquartile range (*N* = 3; *n* = 53 cells (control), *n* = 47 cells (CK666)). Data were analyzed by one-way Mann–Whitney test; ***P < 0.0001. **e**, Left, representative images of DCs from LmnA/C^WT^ and LmnA/C^KO^ DCs. CCR7 is in gray, and the nuclei are in red; scale bar, 5 µm. Right, box plots showing the minimum to maximum range of CCR7 intensity (*N* = 3; *n* = 54 cells (LmnA/C^WT^), *n* = 60 cells (LmnA/C^KO^)). Data were analyzed by ordinary one-way ANOVA; ****P < 0.0001. **f**, Left, representative images of DCs from LmnA/C^WT^ and LmnA/C^KO^ DCs. cPLA_2_ is in gray, and the nuclei are in red; scale bar, 5 µm. Right, box plot showing the minimum to maximum range of cPLA_2_ intensity (*N* = 3; *n* = 141 cells (LmnA/C^WT^), *n* = 144 cells (LmnA/C^KO^)). Data were analyzed by unpaired *t*-test with a Welch’s correction; ****P < 0.0001. Source data

To investigate the molecular basis of this mechanism, we analyzed whether ARP2/3 controls the folding and tension of the nuclear envelope, as we previously showed that these were critical for confinement-induced cPLA_2_ activation in HeLa cells^21^. Staining of the nuclear envelope using the lamina-associated polypeptide 2 (LAP2) marker showed that it was less folded in DCs confined at 3 µm than in DCs confined at 4 µm (Fig. 4a). Noticeably, this difference was abrogated when treating DCs with CK666. Quantification of nuclear envelope folding by measuring the excess of the nuclear envelope perimeter (EOP_NE_) showed that EOP_NE_ was significantly lower in DCs confined at 3 µm and increased after inhibition of ARP2/3 (Fig. 4b).These results suggest that branched actin nucleation is required for confinement-induced unfolding of the nuclear envelope. This finding was further confirmed when analyzing the elliptic Fourier decomposition (EFC) ratio of the nuclear envelope, which compares the relative contribution of the first-order ellipse (perfect and smooth) to the contribution of subsequent-order ellipses required to fit shape irregularities. Indeed, CK666 treatment of DCs confined at a height of 3 µm decreased the EFC ratio, indicating that the nuclear envelope of ARP2/3-inhibited DCs exhibits a more irregular shape than that in untreated confined DCs (Fig. 4c). Collectively, these data strongly suggest that ARP2/3-dependent actin nucleation is needed to unfold the nuclear envelope of DCs in response to cell shape changes.

Nuclear envelope tension can be measured using a membrane tension sensor targeted to the endoplasmic reticulum (ER) membrane (ER Flipper-TR) detected by fluorescence lifetime imaging (FLIM)^36,37^. Unfortunately, DCs failed to take up this sensor probe. We therefore turned to HeLa cells, which were initially used to show that increasing nuclear envelope tension led to cPLA_2_ activation^22^. Remarkably, we observed that treating HeLa cells with CK666 diminished nuclear envelope tension, as shown by a decreased lifetime of FLIM signal (Fig. 4d). To provide further evidence of nuclear envelope tension being needed for cPLA_2_ activation in DCs, we used DCs lacking lamin A/C (*Lmna*-knockout (LmnA/C^KO^) cells), which cannot build up nuclear envelope tension in response to deformation^21,38^. We found that LmnA/C^KO^ DCs did not upregulate CCR7 expression nor translocate cPLA_2_ into the nucleus when confined at a height of 3 µm (Fig. 4e,f). These results highlight that ARP2/3 facilitates unfolding and tensioning of the nuclear envelope in response to cell shape sensing in DCs, thereby tuning cPLA_2_ activation.

### Shape sensing tunes steady-state migration of DCs to lymph nodes

So far, we have shown that WASp–ARP2/3-dependent actin remodeling in response to shape changes defines the activation threshold of cPLA_2_ and the capacity of DCs to upregulate the two processes required for migration to lymph nodes, specifically cell motility and CCR7 expression. These data strongly suggest that (1) WASp–ARP2/3-dependent cPLA_2_ activation in response to cell shape changes might license DCs for migration to lymph nodes in the absence of inflammation, and (2) by restraining the activation of this shape-sensing pathway, Arpin might act as a negative regulator of this process in vivo. Such a regulatory mechanism could limit the number of DCs that acquire CCR7 expression and migrate to lymph nodes as a result of shape sensing during environment patrolling, thereby increasing their time of tissue residency.

**Fig. 5:**
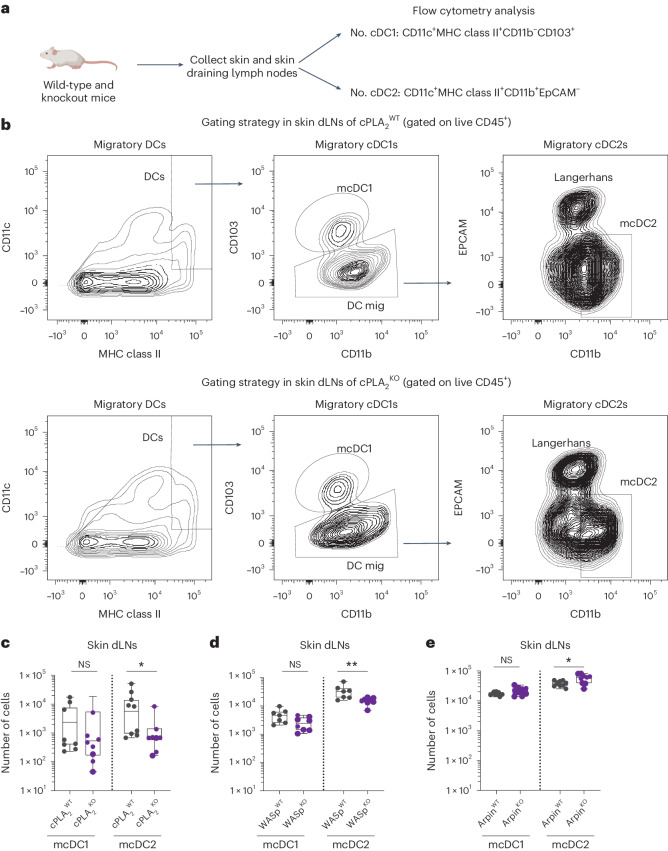
Steady-state migration of skin migratory cDC2s is cell shape sensitive. **a**, Schematic representation of the work flow. The picture of the mouse was created using BioRender.com. **b**, Gating strategy to quantify DCs in skin draining lymph nodes (dLNs). Top, representative example of the data in cPLA_2_^WT^ inguinal lymph nodes. Bottom, representative example of the data obtained in cPLA_2_^KO^ inguinal lymph nodes. Briefly, after gating on live cells, immune cells were identified as CD45^high^; CD11c and MHC class II were then used to differentiate lymph node-resident DCs (MHC class II^low^CD11c^high^) from migratory (mig) DCs (MHC class II^high^CD11c^high^). Among migratory DCs, migratory cDC1s (mcDC1) were identified as CD11b^low^CD103^high^, and migratory cDC2s (mcDC2) were identified as CD11b^high^EPCAM^low^. **c**, Box plots showing the minimum to maximum range in log scale of the number of migratory DCs in cPLA_2_^WT^ and cPLA_2_^KO^ mice (*N* = 3 independent experiments, where each dot is one mouse). Data were analyzed by one-way Mann–Whitney *U*-test; *P = 0.0221. **d**, Box plots showing the minimum to maximum range in log scale of the number of migratory DCs in WASp^WT^ and WASp^KO^ mice (*N* = 3 independent experiments, where each dot is one mouse). Data were analyzed by ordinary one-way Mann–Whitney *U*-test; **P = 0.0059. **e**, Box plots showing the minimum to maximum range in log scale of the number of migratory DCs in Arpin^WT^ and Arpin^KO^ (*N* = 3 independent experiments, where each dot is one mouse). Data were analyzed by ordinary one-way Mann–Whitney *U*-test; *P = 0.04. Source data

**Extended Data Fig. 4:**
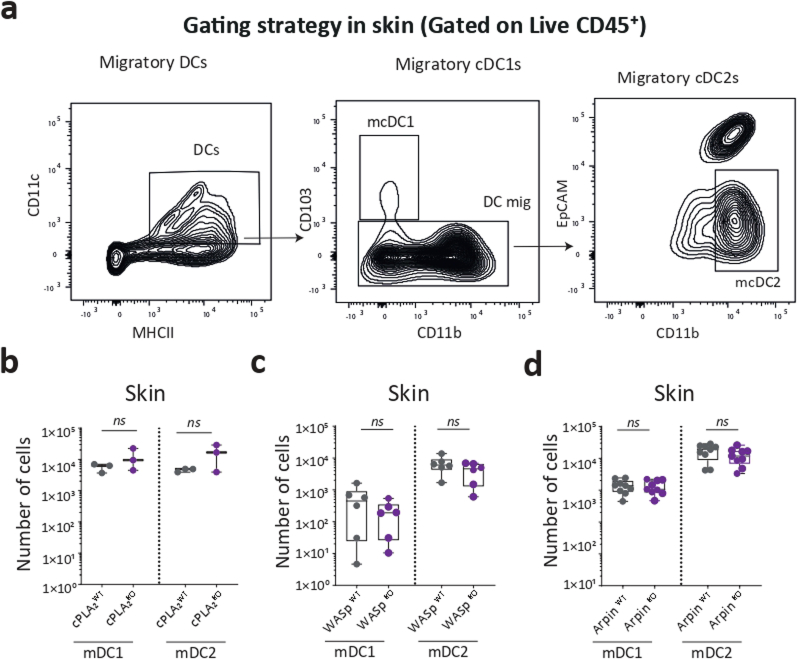
Gating strategy for DC quantification in the skin. (**a**) Gating strategy to quantify DCs in the skin of different mice at steady-state: after gating on live cells, immune cells were identified as CD45^high^; CD11c and MHCII were then used to differentiate lymph node-resident DCs (MHCII ^low^, CD11c^high^) from migratory DCs (MHCII^high^, CD11c^high^). Among migratory cDCs, mDC1s were identified as CD11b^high^, CD103^low^ and mDC2s as CD11b^high^, EPCAM^low^. (**b**) Box plots with min to max range in Log scale of the number of DCs in the skin of cPLA_2_^WT^ and cPLA_2_^KO^ mice, N = 2 independent experiments, n = 3 mice (where each dot is a mouse).Mann-Whitney *U*-test ns: non-significant. (**c**) Box plots with min to max range in Log scale of the number of migratory DCs in the skin of WASp ^WT^ and WASp ^KO^ mice, N = 2 independent experiments, n = 6 mice (where each dot is a mouse).Mann-Whitney *U*-test, ns: non-significant. (**d**) Box plots with min to max range in Log scale showing the number of migratory DCs in the skin of Arpin ^WT^ and KO mice, N = 3 independent experiments, n = 9 mice (where each dot is a mouse). Mann-Whitney *U*-test, ns: non-significant. Source data

To assess the physiological relevance of shape sensing by ARP2/3 and cPLA_2_, we evaluated whether cPLA_2_, WASp and Arpin deficiencies altered the number of migratory DCs present in skin-draining lymph nodes at steady state by flow cytometry. Conventional skin DCs can be divided into the following two main subtypes based on surface marker expression: type 1 conventional DCs (cDC1s) and type 2 conventional DCs (cDC2s)^39^. Although both populations can migrate from the skin to the lymph nodes, the migration rates of cDC2s have been found to be more elevated than the migration rates of cDC1s at steady state^39^. Strikingly, we observed that the numbers of migratory cDC2s found in inguinal lymph nodes were significantly decreased in WASp^KO^ and cPLA_2_^KO^ mice (Fig. 5a–d). No such difference was detected for migratory cDC1s, which, as expected from prior findings, were less represented in these secondary lymphoid organs under homeostatic conditions. Conversely, Arpin^KO^ mice displayed enhanced numbers of migratory cDC2s in lymph nodes compared to their wild-type counterparts (Fig. 5e). Of note, analysis of DC numbers in the skin of these animals showed no significant difference (Extended Data Fig. 4a–d), excluding the possibility that the differences observed in lymph node cDC2 numbers could result from altered cDC2 development and/or survival in the skin of WASp^KO^, cPLA_2_^KO^ or Arpin^KO^ mice. Hence, ARP2/3 activity controlled by WASp and Arpin finely tunes the number of DCs that migrate to lymph nodes at steady state, possibly by controlling the cPLA_2_ activation threshold and downstream CCR7 expression in response to shape sensing. Of note, although WASp deficiency was not found to decrease DC motility in confinement ex vivo^34^, we cannot exclude that in vivo, it could contribute to DC migration to lymph nodes by other means than activating cPLA_2_ (refs. ^34,40^). Together, these data suggest that steady-state migration of DCs to lymph nodes might result, at least in part, from the events of shape changes that they experience while patrolling the complex environment of the skin, suggesting a contribution of cell mechanics to this process.

### IKKβ and NF-κB activity control the ARP2/3–cPLA_2_ shape-sensing axis

The cDC2s that migrate from the skin to the lymph nodes at steady state were shown to display a specific transcriptional profile enriched for NF-κB- and type I interferon (IFN)-related genes^39^. NF-κB activation downstream of the IKKβ (*Ikbkb*) kinase was further described as required for the migration of DCs from the skin to the lymph nodes at steady state and after inflammation^41^. Of note, this pathway is the only pathway described so far as being implicated in the homeostatic migration of skin DCs. We therefore investigated whether the ARP2/3–cPLA_2_ shape-sensing axis relies on IKKβ-dependent NF-κB activation and whether it triggers global transcriptional reprogramming of DCs. To this end, we compared the transcriptomes of cPLA_2_^WT^ and cPLA_2_^KO^ DCs confined at a height of 3 µm by bulk RNA sequencing (RNA-seq); nonconfined cells were used as negative controls.

**Fig. 6:**
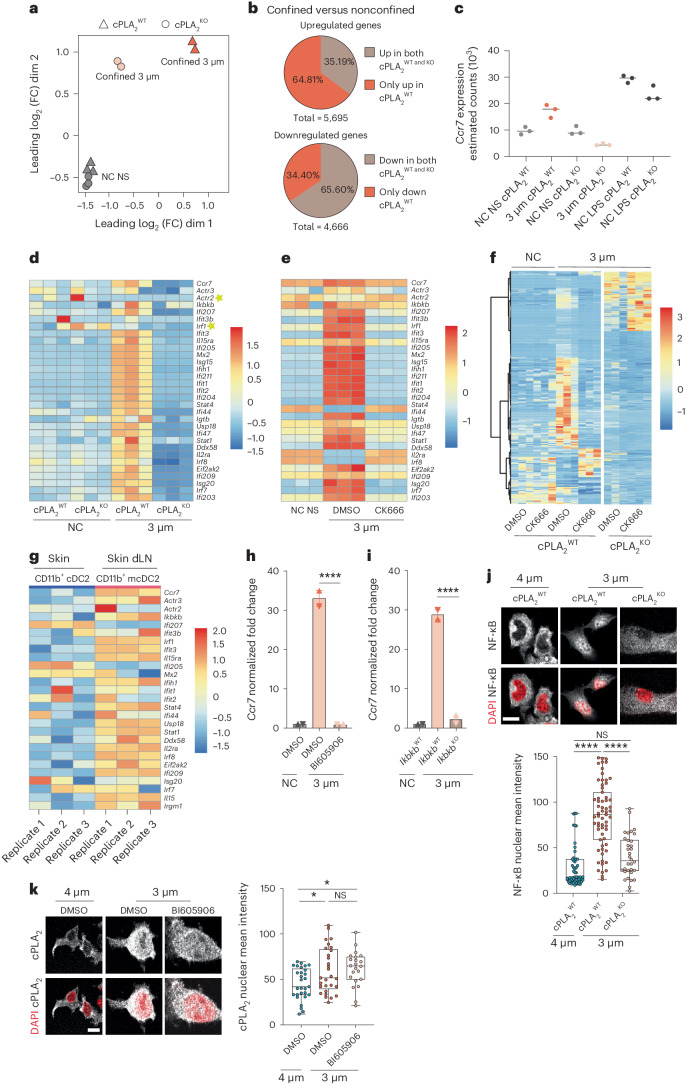
cPLA_2_ reprograms DC transcription in an IKKβ–NF-κB-dependent manner. **a**–**d**, Bulk RNA-seq analysis of cPLA_2_^WT^ and cPLA_2_^KO^ DCs; NC NS, nonconfined nonstimulated. **a**, Multidimensional scaling (MDS) of the samples. Sample groups under different confinement conditions are represented by different forms. Each dot represents a biological replicate; dim, dimension; FC, fold change. **b**, Pie charts showing proportions of differentially expressed genes in cells under confined conditions compared to nonconfined cells (false discovery rate < 0.05 and log_2_ (fold change) of <–1.0 or >1.0). Top, number of upregulated genes in confinement in cPLA_2_^WT^ and cPLA_2_^KO^ DCs (5,695 genes). Bottom, number of downregulated genes in confinement in both cPLA_2_^WT^ and cPLA_2_^KO^ DCs (4,666 genes). **c**, *Ccr7* estimated gene counts in the different samples/conditions. **d**, Heat map of genes harboring a similar expression pattern as *Ccr7*. **e**, Heat map of the genes moving with *Ccr7* expression in response to treatment with CK666 (30 µM). **f**, Heat map of all differentially expressed genes in response to treatment with CK666 (30 µM) in confined and nonconfined DCs. **g**, Heat map showing normalized expression of some IFN-stimulated genes in the CD11b^+^ cDC2 subset from the dermis and their migratory counterparts in the cutaneous draining lymph node of healthy mice (GSE49358 microarray study from Tamoutounour et al.^39^). **h**, *Ccr7* expression in response to treatment with BI605906 (30 µM). Data are shown as mean ± s.d. (*N* = 2, *n* = 6) and were analyzed by ordinary one-way ANOVA; ****P < 0.0001. **i**, *Ccr7* expression in cells from *Ikbkb*^WT^ or *Ikbkb*^KO^ mice. Data are shown as mean ± s.d. (*N* = 2, *n* = 6) and were analyzed by ordinary one-way ANOVA; ****P < 0.0001. **j**, Top, representative images of cPLA_2_^WT^ and cPLA_2_^KO^ DCs. NF-κB (P65) is in gray, and the nuclei are in red; scale bar, 10 µm. Bottom, box plot showing the minimum to maximum range of NF-κB (P65) intensity (*N* = 2, *n* = 55 cells in 4 µm (cPLA_2_^WT^), *n* = 63 cells in 3 µm (cPLA_2_^WT^), *n* = 34 cells in 4 µm (cPLA_2_^KO^)). Data were analyzed by Kruskal–Wallis test; ****P < 0.0001. **k,** Left, representative images of DCs treated with the IKKβ inhibitor BI605906 (30 µM) and nontreated control DCs. cPLA_2_ is in gray, and nuclei are in red; scale bar, 12 µm. Right, box plot showing the minimum to maximum range of cPLA_2_ intensity (*N* = 2, *n* = 29 cells (DMSO), *n* = 23 cells (BI605906)). Data were analyzed by ordinary one-way ANOVA; *P = 0.0175. Source data

Principal-component and clustering analyses revealed that although nonconfined nonstimulated cPLA_2_^WT^ and cPLA_2_^KO^ samples clustered together, this did not apply to confined cPLA_2_^WT^ and cPLA_2_^KO^ cells, showing that they display important differences in their gene expression profiles (Fig. 6a). These results indicate that cPLA_2_ impacts the transcriptomes of confined DCs but has no major effect on nonconfined cells at steady state. More specifically, we observed that ~5,000 and ~4,600 genes were up- and downregulated, respectively, in cPLA_2_^WT^ DCs confined at a height of 3 µm compared to under nonconfined conditions (Fig. 6b). Comparison of cPLA_2_^WT^ and cPLA_2_^KO^ DCs showed that more than half of the genes upregulated by confinement relied on cPLA_2_ (~3,600 genes; Fig. 6b). Consistent with our findings, *Ccr7* upregulation was found to be fully lost in confined cPLA_2_^KO^ DCs, which showed decreased *Ccr7* mRNA levels after confinement (Fig. 6c). Strikingly, among the 103 genes following the same expression pattern as *Ccr7* were 2 genes encoding the major subunits of ARP2/3 (*Actr2* and *Actr3*), the gene encoding IKKβ (*Ikbkb*) and several IFN-stimulated genes (Fig. 6d). Consistently, RNA-seq analysis of DCs confined at a height of 3 µm and treated with CK666 showed that the genes that were upregulated in expression after confinement in a cPLA_2_-dependent manner, including the 103 genes behaving similar to *Ccr7*, were also dependent on ARP2/3 activity (Fig. 6e,f). Of note, CK666 treatment showed additional effects on gene expression similar to the cPLA_2_^KO^ condition (Fig. 6f), indicating that ARP2/3 regulates transcription in response to confinement by additional means than activating cPLA_2_.

Importantly, direct comparison of the transcriptional profiles of our confined bone marrow-derived DCs with the transcriptional profile described for skin migratory cDC2s revealed that they exhibited similar signatures (genes following the *Ccr7* expression pattern; Fig. 6g). These results suggest that the ARP2/3–cPLA_2_ shape-sensing axis imprints DCs with a similar transcriptional program as the program displayed by skin cDC2s migrating to lymph nodes at steady state, confirming the in vivo contribution of this mechanical pathway. These results prompted us to investigate whether cPLA_2_ and IKKβ–NF-κB were part of the same signaling route. We found that (1) confinement at a height of 3 µm did not lead to *Ccr7* upregulation in DCs treated with the IKKβ inhibitor BI605906 or in DCs lacking the *Ikbkb* gene (Fig. 6h,i), and (2) NF-κB nuclear translocation was compromised in confined DCs lacking cPLA_2_ (Fig. 6j). By contrast, IKKβ inhibition had no effect on nuclear accumulation of cPLA_2_ in confined DCs (Fig. 6k). These data strongly suggest that cPLA_2_ acts upstream of IKKβ and NF-κB to trigger upregulation of CCR7 expression after shape sensing in DCs. Together, our data support a model where shape sensing through the ARP2/3–cPLA_2_ axis activates the IKKβ–NFκB pathway and thereby licenses DCs to migrate to lymph nodes at steady state.

### Shape sensing endows DCs with specific immunoregulatory properties

**Fig. 7:**
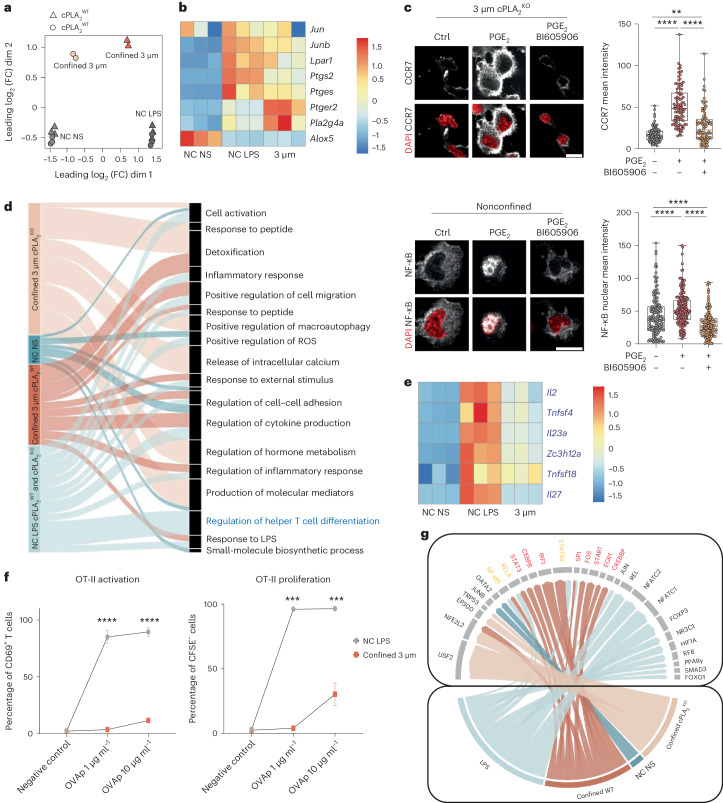
cPLA_2_-dependent transcriptional reprogramming in response to shape sensing shapes DC properties. **a**, MDS of samples. **b**, Heat map of cPLA_2_-related genes. **c**, Top, representative images of untreated control cPLA_2_^KO^ DCs and cPLA_2_^KO^ DCs treated with 2.5 µg ml^–1^ PGE_2_ for 30 min in the presence or absence of BI605906 (30 µM) before confinement. CCR7 is in gray, and nuclei are in red; scale bar, 10 µm; Gaussian blur of 0.5 µm. Right, box plot showing the minimum to maximum range of CCR7 intensity (*N* = 3; *n* = 87 cells in 3 µm (control), *n* = 99 cells in 3 µm (PGE_2_), *n* = 87 cells in 3 µm (PGE_2_ + BI605906)). Data were analyzed by Kruskal–Wallis test; ****P < 0.0001; **P = 0.0026. Bottom, representative images of nonconfined untreated DCs or DCs treated with 2.5 µg ml^–1^ PGE_2_ for 30 min in the presence or absence of BI605906 (30 µM). NF-κB (P65) is in gray, and nuclei are in red; scale bar, 10 µm; Gaussian blur of 0.5 µm. Right, box plot showing the minimum and maximum range of NF-κB intensity (*N* = 3; *n* = 133 cells (control), *n* = 191 cells (PGE_2_), *n* = 138 cells (PGE_2_ + BI605906)). Data were analyzed by Kruskal–Wallis test; ****P < 0.0001. **d**, Pathway analysis of differentially expressed genes in confined versus LPS-treated DCs; arrow width corresponds to the enrichment score from the significantly enriched (*P* < 0.05) genes. **e**, Heat map of genes in the ‘regulation of helper T cell differentiation’ pathway marked in blue in **d**. **f**, Antigen presentation assays with T cells incubated with DCs activated with LPS or confined at a height of 3 µm. Left, percentage of CD69^+^CD4^+^ T cells among live cells after 18 h of incubation with OVA peptide II (OVAp). Right, Percentage of CFSE^–^CD4^+^ OT-II T cells among live cells after 3 days of incubation with OVA peptide II. Data are shown as mean ± s.e.m. of duplicate measures of each independent experiment (*N* = 5). Data were analyzed by multiple unpaired Student’s *t*-tests; ***P = 0.0001; ****P < 0.0001. **g**, Transcription factor analysis of the different conditions. Transcription factor activity estimation used the TRUUST database, which predicts transcription factor activity and assigns a score. Arrow thickness corresponds to the enrichment score of each transcription factor. Source data

**Extended Data Fig. 5:**
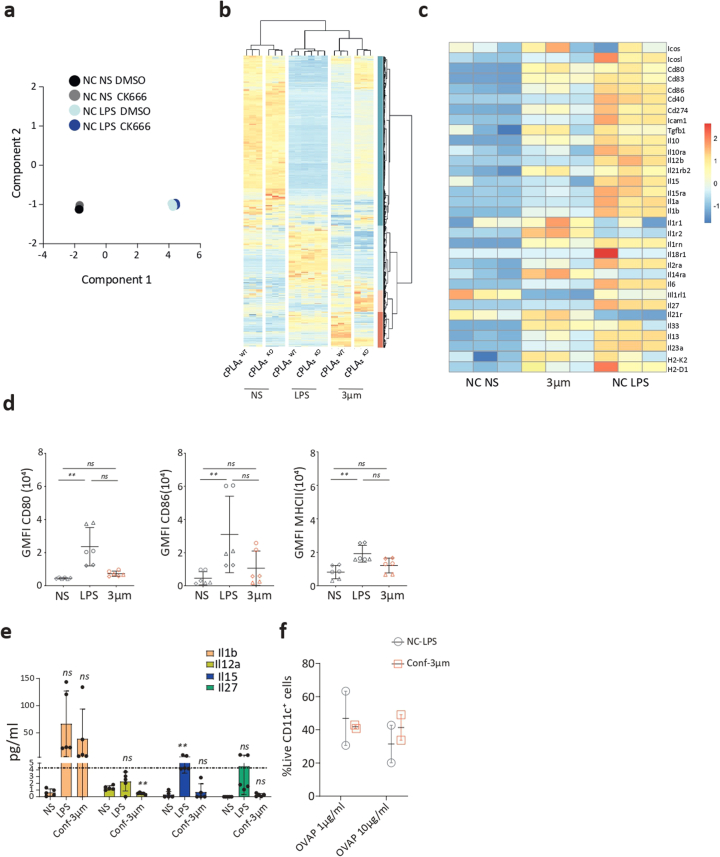
Transcriptional changes in DC in response to confinement are cPLA2-dependent. (**a**) Multidimensional scaling (MDS) of non-confined DCs stimulated or not with LPS and treated or not with CK666 (**b**) Heatmap of all the differentially expressed genes in cPLA_2_ WT and KO cells in all the different conditions (**c**) Heat map of examples of cytokines and costimulatory and MHC-I genes in DCs in response to confinement or LPS (**d**) FACS analysis of some immune-activating genes expressed by DCs not-stimulated (NS) controls or in response to LPS or confinement at 3 µm height. Graphs of Mean with SD showing geometric mean intensity of CD80, CD86, and MHCII. N = 3 each condition was done in duplicates, Kruskal-Wallis test CD80 plot: ** p = 0.003, ns: not significant p = 0.1547. CD86 plot: **: p = 0.0083, ns: not significant p = 0.4684. MHCII plot: **: p = 0.0041, ns: not significant p = 0.2886 (**e**) cytokine secretion analysis by luminex of some cytokines in the supernatant of LPS or confined DCs 48h after the confinement or the LPS activation. Graph of Mean with SD of 5 independent experiments, each condition was done in triplicates. 2 way ANOVA test **: p = 0.0039, ns: not significant p = 0.1186, p = 0.3014, p = 0.3329, p = 0.7488, p = 0.1212, p = 0.0922 respectively(**f**) percentage of live DCs CD11c high after 48 hour of confinement and incubation with OTII T-cells. Graph: mean with SEM, n = 6, N = 2 independent experiments, each condition was done in duplicates. Source data

DC migration to lymph nodes at steady state helps maintain peripheral tolerance^7,9,42^. This is in sharp contrast to microbe-induced DC migration to lymph nodes that leads to activation of T cells capable of fighting these infectious agents. Our results show that ARP2/3 and cPLA_2_ are required for CCR7 upregulation in response to confinement but not in response to LPS (Extended Data Figs. 2d and 3c). Similarly, CK666-treated DCs clustered together, suggesting minor differences in their transcriptional profiles whether or not they were activated with LPS (Extended Data Fig. 5a). This suggests that the shape-sensing pathway described here might be specifically involved in steady-state, rather than microbe-induced, migration of DCs to lymph nodes. This scenario would be particularly appealing as no specific mechanism has been identified so far for the triggering of homeostatic DC migration, the IKKβ–NF-κB pathway being required for DC migration to lymph nodes both at steady state and after inflammation^41^. To test this hypothesis, we compared the transcriptomes of DCs expressing or not expressing cPLA_2_ either confined at a height of 3 µm or treated with LPS. Strikingly, principal-component and clustering analyses revealed that cPLA_2_ had no substantial impact on the global gene expression pattern induced by microbial stimulation (Fig. 7a and Extended Data Fig. 5b), confirming the specific requirement of cPLA_2_ for mechanical reprogramming of DCs. Interestingly, while some genes associated with the cPLA_2_ pathway were upregulated under both conditions, others, including the genes encoding cPLA_2_ and the prostaglandin E_2_ (PGE_2_) receptor, were more induced in confined DCs than in LPS-treated cells (Fig. 7b). This result prompted us to investigate whether production of PGE_2_ from AA could contribute to DC transcriptional reprogramming by confinement. We found that the addition of PGE_2_ to confined cPLA_2_^KO^ DCs led to upregulation of CCR7 expression (Fig. 7c), showing that PGE_2_ can compensate cPLA_2_ deficiency. This was not observed when inhibiting IKKβ (Fig. 7c). Accordingly, PGE_2_ was found to induce NF-κB nuclear translocation (Fig. 7c). Together, these data suggest that DC transcriptional reprogramming after shape sensing involves prostaglandin production downstream of cPLA_2_, which in turn activates IKKβ and NF-κB in a paracrine manner.

**Extended Data Fig. 6:**
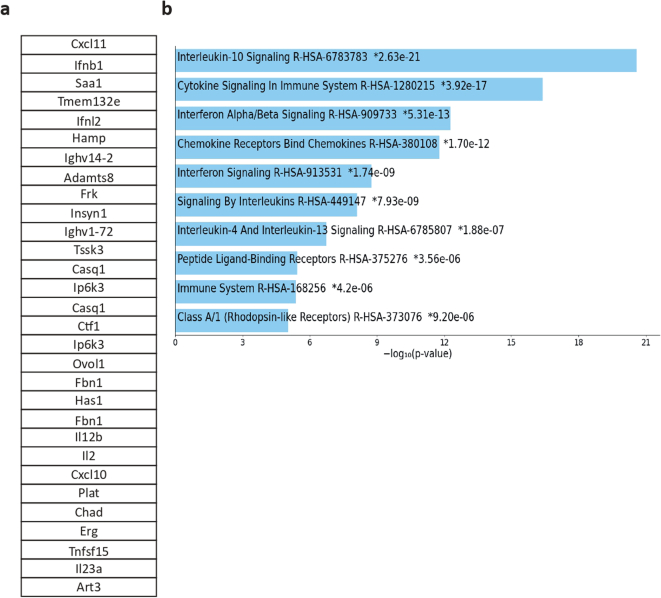
The Arp2/3 cPLA_2_ pathway induces a tolerogenic signature in the confined DCs. (**a, b**) statistical analysis used to determine genes with log FC > 1 and P value < 0.05 is detailed in the methods section. (**a**)Example of the top 30 genes that are upregulated in the confined DCs which their expression depends on cPLA_2_ and Arp2/3 activity. (**b**) Reactom pathway analysis of the genes upregulated in DCs in response to shape sensing in an Arp2/3- cPLA_2_- dependent manner. Source data

To better understand the specificity of DC reprogramming in response to shape sensing, we compared the pathways induced by DC confinement or by LPS (Fig. 7d). One of the most striking differences observed was related to the ‘regulation of helper T cell differentiation’ pathway that was exclusively induced by LPS (Fig. 7d,e). Mounting an efficient T cell response against a microbial threat typically requires the following three signals from DCs: specific antigenic peptide presented on major histocompatibility complex (MHC; signal 1), co-stimulation (signal 2) and cytokines (signal 3). The two latter signals ensure T cell activation rather than tolerization as well as proper proliferation. Accordingly, we observed that genes encoding MHC class I, MHC class II and key co-stimulatory molecules (CD80 and CD86) were expressed at lower levels in confined DCs than in LPS-treated cells (Extended Data Fig. 5c,d). Confined DCs also expressed fewer stimulatory cytokines (interleukin-2 (IL-2), IL-12, IL-15 and IL-27), resulting in lower secretion levels (Extended Data Fig. 5c,e). This suggests that confined DCs might be less potent than LPS-treated cells for T lymphocyte activation. To test this hypothesis, we loaded the two types of DCs with the class II ovalbumin (OVA) antigenic peptide and incubated them with OT-II transgenic T cells. OT-II cell stimulation with DCs that experienced confinement led to both lower T cell activation (quantified as upregulation of CD69) and lower proliferation (quantified by CFSE dilution) than stimulation with LPS-treated cells (Fig. 7f). Cell viability was comparable under both conditions (Extended Data Fig. 5f). These data confirmed that confined DCs were less potent than LPS-treated cells in activating T lymphocytes. In support of these results, when applying a threshold to the 3,000 genes upregulated in confined DCs in an ARP2/3- and cPLA_2_-dependent manner, we obtained a list of 467 upregulated genes mostly related to cytokines/chemokines and dominated by the pathway of the tolerogenic cytokine IL-10 (see Extended Data Fig. 6a,b for the top 30 genes). Together, our data suggest that the ARP2/3–cPLA_2_ shape-sensing pathway can not only tune steady-state migration of DCs to lymph nodes but also further endow them with specific immunoregulatory properties.

To gain insights into the mechanisms accounting for this specificity, we compared the transcription factor binding sites found in the promoters of the genes enriched in DCs confined at a height of 3 µm or treated with LPS. This analysis allows us to infer the nature of the transcription factors differentially implicated in the two types of DC responses. We found that DC confinement activated IRF1-, STAT1-, STAT3- and STAT5A-dependent gene transcription better than LPS (Fig. 7g). These transcription factors are known to be involved in IFN signaling and/or activation of NF-κB^43^, in good agreement with our results showing that these two pathways are enriched following shape sensing (Fig. 6d). Noticeably, IRF1, STAT3 and STAT5A have been implicated in the acquisition of tolerogenic properties by DCs^44,45^. None of these transcription factors were activated in confined cPLA_2_^KO^ DCs (Fig. 7g). Together, our results suggest that the interplay between the cytoskeleton and the lipid metabolism enzyme cPLA_2_ transcriptionally reprograms DCs in response to precise shape changes, endowing them with the ability to reach lymph nodes in an immunoregulatory state that might facilitate the tolerogenic function proposed for these cells at steady state.

## Discussion

Here, we show that DCs are equipped with an exquisitely sensitive shape-sensing mechanism that defines their migratory behavior and immune phenotype. This mechanism requires ARP2/3 nuclear envelope tensioning and translocation of the cPLA_2_ lipid metabolism enzyme into the nucleus. cPLA_2_ in turn activates NF-κB, leading to upregulation of CCR7 expression and DC migration to lymph nodes at steady state. These findings might explain why, despite two decades of research, the signals responsible for homeostatic DC migration from the periphery to the lymph nodes have remained largely unknown. Rather than sensing (bio)chemical signals, DCs might sense the physical constraints they encounter during environment patrolling through this ARP2/3–cPLA_2_–NF-κB shape-sensing pathway. Interestingly, although this mechanical pathway shares signaling players, such as NF-κB, with the pathway induced by microbial sensing, it does not involve the direct engagement of DC receptors such as Toll-like receptors (TLRs) and NOD-like receptors (NLRs) and leads to an overlapping but distinct transcriptional program in these cells.

Previous work from us and others suggests that a specific threshold of nuclear deformation can promote nuclear envelope tensioning and cPLA_2_ insertion into the nuclear membrane, leading to its activation^21,23^. Here, we provide direct evidence for this threshold being tuned by the actin cytoskeleton by showing that (1) ARP2/3 activity promotes nuclear envelope unfolding and tensioning in response to confinement, and (2) confinement induces the appearance of a large actin cloud in the vicinity of the nucleus, probably as a result of cortical actin flow directed toward the cell center, as previously shown in fibroblasts^46^. We propose that flowing cortical actin, when pressed against the nucleus after confinement, exerts forces, potentially through the LINC complex, to unfold the nuclear lamina and tense the nuclear membrane. The increased nuclear/cytosolic ratio of cPLA_2_ observed in DCs confined at a height of 3 μm could be due to its insertion into the tensed inner nuclear envelope. Of note, we do not exclude that ARP2/3-dependent forces might also open nuclear pores to enhance cPLA_2_ translocation from the cytoplasm to the nucleus, similar to what had been shown for YAP/TAZ^47^, thereby contributing to nuclear accumulation of the enzyme.

We postulate that self-activation of the ARP2/3–cPLA_2_ axis originates from the successive events of deformation that DCs undergo as they move through the complex environment of tissues. These deformation events would induce a ‘cellular massage’ and thereby activate shape sensing in DCs. Notably, the ARP2/3–cPLA_2_ pathway is induced at a precise amplitude of cell deformation compatible with the shape changes observed in DCs patrolling the skin (Fig. 1a)^19,48^. Yet, the physical constraints imposed by the environment are likely to vary in distinct tissues^49^ or pathological contexts, such as the tumor environment^50^. Determining whether the range of sensitivity of DCs to deformation is adapted to each tissue would be of the utmost importance to understand how the ARP2/3–cPLA_2_ shape-sensing pathway controls both DC tissue exit to lymph nodes and their immunoregulatory properties. Nonetheless, our results emphasize that the physical properties of tissues indeed contribute to shaping immunity. The ability of immune cells to integrate these physical properties with biochemical environmental cues should be investigated.

Importantly, the ARP2/3–cPLA_2_ shape-sensing pathway is the first pathway identified so far to be specifically involved in homeostatic DC migration to lymph nodes, a process essential for the maintenance of tolerance. We show that this shape-sensing axis has no impact on DC transcriptional reprogramming by microbial components such as LPS. Even though confined and LPS-treated DCs share a considerable part of their transcriptional program, LPS treatment led to activation of specific pathways associated with efficient T cell activation, which were less induced by confinement. Consistently, confined DCs were less efficient in activating T cells than LPS-treated cells. These results are in agreement with the tolerogenic function proposed for homeostatic DC migration to lymph nodes^41,51^. Furthermore, several of the transcription factors specifically activated in confined DCs have been described as associated with immune tolerance^44^, and examining genes that were most upregulated after shape sensing in an ARP2/3–cPLA_2_-dependent manner revealed IL-10 signaling as the predominant pathway. However, we acknowledge that these results only constitute indirect evidence for shape sensing, leading to tolerogenic DCs, and that additional functional and in vivo studies will be required to directly visualize cPLA_2_ activation in tissue-patrolling DCs and to show that these cells can protect mice from autoimmunity.

Interestingly, it has recently been shown that spleen CCR7^+^ tolerogenic DCs can also emerge after internalization of apoptotic bodies, a process referred to as efferocytosis^52^. Along the same line, efferocytosis can trigger a tolerogenic phenotype in CCR7^+^ tumor-infiltrating DCs by regulating cholesterol metabolism^53^. Future studies aimed at determining whether and how the here described shape-sensing pathway functionally interacts with these efferocytosis-dependent pathways should shed light on how the balance between tolerance and immunity is critically tuned by distinct tissue environmental properties.

## Methods

### Mice

For animal care, we strictly followed the European and French National Regulation for the Protection of Vertebrate Animals used for Experimental and other Scientific Purposes (Directive 2010/63; French Decree 2013-118, Authorization DAP number 43530-2023051216135493 v2 given by National Authority). Experiments were performed on 8- to 14-week-old male or female mice. Mice were maintained under specific pathogen-free conditions at the animal facility of Institut Curie, in accordance with institutional guidelines, and housed in a 12-h light/12-h dark environment with free access to water (osmotic water) and food (MUCEDOLA, 4RF25SV Aliment Pellets 8 × 16 mm irradiated 2.5 Mrad and DietGel Recovery, 2 oz/DietGel Boost, 2 oz). Littermates or age-matched mice were used as controls for all experiments involving knockout animals. Breeder mice were previously backcrossed to C57BL/6 mice for seven generations. C57BL6/J mice were obtained from Charles River (000664). CCR7–GFP mice were obtained from Jackson Laboratory (027913) and were bred in our animal facility^30^. *Itgax*-*cre* mice were bred in our animal facility^54^. *Arpin-* and *Pla2g4a-*conditional-knockout mice were generated by Centre d’Immunophénomique using CRISPR–Cas9 technology and were crossed in our animal facility with *Itgax-cre* mice. *Ikbkb-*knockout mice were from the laboratory of T. Lawrence (Centre d’Immunologie de Marseille-Luminy, Marseille, France), as described in Baratin et al.^41^. WASp^KO^ mice on a C57BL/6 (CD45.2) genetic background were from the laboratory of F.B. Experiments were performed using homozygous WASp^–/–^ mice as knockout animals. *Lmna*^fl/fl^*Vav1-cre*^+/−^ (lamin A/C CK^55,56^)mice were obtained from the laboratory of N.M. YFP–CD11c mice were from the laboratory of S. Amigorena (Institute Curie, U932, Paris, France)^57^.

### Cells

DCs were obtained following a protocol adapted by the Ricciardi-Castagnoli and Amigorena labs. Using this differentiation protocol >90% of the cells recovered after 10 days of culture are positive for the CD11c marker, in addition to MHC class II^58,59^. In brief, both whole legs from 6- to 8-week-old mice were flushed to obtain bone marrow. Cells were maintained in culture for 10 days in IMDM (Sigma-Aldrich) containing decomplemented and filtered 10% fetal bovine serum (FBS; Biowest), 20 mM l-glutamine (Gibco), 100 U ml^–1^ penicillin–streptomycin (Gibco), 50 μM 2-mercaptoethanol (Gibco) and 50 ng ml^–1^ granulocyte–macrophage colony-stimulating factor containing supernatant obtained from transfected J558 cells tested by enzyme-linked immunosorbent assay. At days 4 and 7 of culture, cells were detached using PBS-EDTA (5 mM) and replated at 0.5:10^6^ cells per ml of medium. The obtained cells were then used at day 10 or 11 as immature cells. This differentiation protocol also promotes the differentiation of granulocytes and macrophages, but they can be separated from DCs based on their adhesion. Granulocytes are eliminated during differentiation because they are nonadherent, whereas macrophages are much more adherent than the other two cell types and stick to the bottom of the plate. The semiadherent fraction of cells, which corresponds to DCs, was thus recovered at day 10 or 11.

#### DC maturation

Day 10 cells were stimulated with 100 ng ml^–1^ LPS (*Salmonella enterica* serotype Typhimurium; Sigma) for 25 min, washed three times with complete medium, replated in fresh medium, left overnight in the incubator and used.

### Six-well plate confiner

Confinement was performed using a six-well plate confiner as previously described^26^.To make the polydimethylsiloxane (PDMS; RTV615) pillars at a certain height, 12-mm glass coverslips were sonicated in methanol, washed in ethanol, plasma treated and placed on the top of a PDMS mixture on top of wafer molds that contained the pillars at the desired height. The PDMS mixture was composed of PDMS A:cross-linker B at a ratio of 1:10 (wt/wt). After adding the coverslips to the wafers, the wafers were baked at 95 °C for 15 min and carefully removed using isopropanol. The wafers were then washed again with isopropanol, dried and plasma treated for 2 min. Next, the molds were incubated with nonadhesive pLL-PEG (SuSoS, PLL (20)-g [3.5]-PEG (2)) at 0.5 mg ml^–1^ in 10 mM HEPES (pH 7.4) buffer for 1 h at room temperature. The coverslips were washed well with water to remove all remaining PEG and incubated in cell medium for 2 h before confinement. To perform the confinement steps, we used a modified version of a classical six-well plate cover; large PDMS pillars were stuck on the coverlid. Six-well plates with a glass bottom were used for cell imaging (MatTek, P06G-1.5-20-F).

### Live imaging experiments

Live timelapse recordings were acquired by Nikon video microscopy with a ×20/0.75-NA dry objective for 4–6 h at 37 °C with 5% CO_2_ atmosphere, by confocal microscopy (Leica DMi8, SP8 scanning head unit) with a ×40/1.3-NA oil objective with a resolution of 1,024 × 1,024 pixels or by inverted spinning desk confocal microscopy with a ×60/1.4-NA oil objective. All microscopes were controlled by Meta Morph software. Images were processed using ImageJ software^60^ (NIH, http://rsb.info.nih.gov/ij/index.html), and analyses were performed using the same software. The following drugs and reagents were used for live imaging experiments: NucBlue (Hoechst33342; DNA marker; Thermo Fischer, R37605), AACOCF3 (selective phospholipase A_2_ inhibitor; Tocris, 1462-5), CK666 (selective ARP2/3 inhibitor; Tocris, 3950), BI605906 (selective IKKβ inhibitor; Tocris, 53001) and PGE_2_ (Stem Cell, 72372).

For GFP quantification, imaging was performed using both phase contrast and GFP channels. Images corresponding to each time point of interest were collected for each condition, the outline of each cell was drawn by hand on the *trans* images, and the mean GFP signal was calculated on the corresponding GFP image using a homemade macro. Of note, only cells with an intensity higher than the background were plotted.

### Lentivirus transduction

Transduced DCs were obtained by transfection of bone marrow-derived DCs. Bone marrow-derived DCs were plated at a concentration of 1 million cells per 2 ml of medium. On day 4, 40 ml of fresh pTRIP-SFFV-GFP-NLS lentivector supernatant was loaded in Ultra-Clear Centrifuge tubes (Beckman Coulter) and ultracentrifuged at 100,000*g* in a SW32 rotor (Beckman Coulter) for 90 min at 4 °C and resuspended in 400 µl of DC medium. Ultracentrifuged virus (200 µl) was used to infect one well of cells in the presence of 8 µg ml^–1^ protamine. Cells were then left for 48 h, washed to remove the viral particles and incubated in new medium until day 10 of culture. For the NLS–GFP construct, pTRIP-SFFV-EGFP-NLS was generated introducing the SV40 NLS sequence (PKKKRKVEDP) by overlapping PCR at the C terminus of GFP in pTRIP-SFFV.

### Immunofluorescence microscopy

After removing the confinement, samples were fixed directly with 4% paraformaldehyde for 30 min, permeabilized with 0.2% Triton X-100 and incubated overnight with primary antibodies at 4 °C. The next day, samples were washed with PBS and incubated with the corresponding secondary antibodies for 1 h, washed three times with PBS and mounted with Fluoromount solution. Imaging was performed using a confocal microscope (Leica DMi8, SP8 scanning head unit) with a ×40/1.3-NA oil objective and a resolution of 1,024 × 1,024 pixels. The following primary antibodies were used for the immunofluorescence stainings: anti-CCR7 (Abcam, ab32527; 1:100), anti-cPLA_2_ (Cell Signaling (Ozyme), 2832; 1:100), anti-NF-κB P65 (Cell Signaling, mAB 8242; 1:200), anti-LAP2 (BD Biosciences, 611000; 1:200) and Alexa Fluor-coupled phalloidin (Invitrogen; 1:300). The following secondary antibodies were used: Alexa Fluor 647 goat anti-rabbit (Invitrogen, A21244; 1:300) and Alexa Fluor 488 goat anti-mouse Fab_2_ (Invitrogen, A11017; 1:300).

#### Calculation of fluorescence intensity

Images were acquired as *z* stacks with a step size of 0.33 µm for each position. Using a homemade macro, the plane of the nucleus was used to quantify the florescence intensity by making a mask on both the nucleus (DAPI channel) and the cell (phalloidin channel).

### Transfection and short interfering RNA (siRNA)

Bone marrow-derived DCs at day 7 (3 × 10^6^) were transfected with 100 μl of Amaxa solution (Lonza) containing siRNA (control or target specific) following the manufacturer’s protocol. Cells were further cultured for 48 h. The following SMARTpool siRNAs were used: ON-TARGETplus *Pla*_*2*_*g4a* siRNA (Dharmacon, L-009886-00-0010) and ON-TARGETplus Non-Targeting Control Pool (Dharmacon, D-001810-10-20).

### RT–qPCR

After removing the confinement, lysis buffer was added directly to recover the confined cells. RNA extraction was performed using an RNeasy Micro RNA kit (Qiagen) according to the manufacturer’s protocol. cDNA was produced using a high-capacity cDNA synthesis kit (Thermo Fisher), according to the manufacturer’s protocol, starting from 1 μg of RNA. qPCR experiments were performed using Taqman Gene Expression Assays (Applied Biosystems) on a Lightcycler 480 (Roche) using the settings recommended by the manufacturer. The following primers were used: Mm99999130_s1 for *Ccr7*, Mm01284324_m1 for *Pla*_*2*_*g4a* and Mm99999915 for *Gapdh* (endogenous control). The expression of each gene of interest was assessed in immature nonconfined cells. Samples were run in triplicate for each condition. Data were subsequently normalized to *Gapdh* values. Values obtained in control immature cells were used as a base unit equal to 1, thus allowing for display of the data as ‘fold greater’ than immature cells. Fold change was calculated using the formula $2^{-\Delta\Delta C_{\mathrm{t}}}$.

### Flow cytometry

After confining the cells, the confiner was removed, and cells were recovered directly by gently washing with medium. Cells were resuspended in buffer (PBS supplemented with 1% bovine serum albumin and 2 mM EDTA). After blocking with Fc antibody (BD Biosciences, 553142; 1:1,000) and processing with a live/dead staining kit (Thermo Fischer, L34966) for 15 min, cells were stained with the desired antibodies for 20 min (at 37 °C for CCR7 staining and at 4 °C all other antibodies). Cells were then washed three times and resuspended in staining buffer. Flow cytometry was performed on an LSRII flow cytometer (BD Biosciences), and data were analyzed using FlowJo software version 10. Mean florescence values of each condition were plotted with GraphPad Prism version 8. The following antibodies were used: anti-CD80 (BD Biosciences, 553769, clone 16-10A1; 1:100), anti-MHC class II (Ozyme, BLE107622, clone M5/114.15.2; 1:400), anti-CD86 (Biolegend, 105037, clone GL-1; 1:200) and anti-CD11c (BD Biosciences, 550261, clone HL3; 1:200) with the corresponding isotypes to each antibody.

For the T cell presentation assay, OVA peptide (Cayla, vac-isq) was added to each condition with the corresponding concentration. Cells were then confined, recovered and counted. T cells were purified from OT-II mice, stained with CFSE and added to the recovered DCs at a ratio of 10:1, respectively. Cells were plated in round-bottom, 96-well plates. OT-II T cell activation was analyzed 18 h later, and after 3 days, proliferation was measured by flow cytometry. Cells were stained in 2 mM EDTA and 5% FBS in PBS following the same protocol as described earlier. Flow cytometry was performed on an LSRII flow cytometer (BD Biosciences), and data were analyzed using FlowJo software version 10. Percentage values were plotted with GraphPad Prism version 8. The following antibodies were used: anti-CD4 (BD Biosciences, 553051, clone RM4-5; 1:100), anti-CD69 (eBioscience, 48-0691-82, clone H1.2F3; 1:300) and anti-TCR (BD Biosciences, 553190, clone MR9-4; 1:300).

### RNA-seq

After removing the confinement, lysis buffer was added directly to recover the confined cells. RNA extraction was performed using an RNeasy Micro RNA kit (Qiagen), according to the manufacturer’s protocol. RNA-seq libraries were prepared from 300 ng to 1 µg of total RNA using an Illumina TruSeq Stranded mRNA Library Preparation kit and an Illumina Stranded mRNA Prep Ligation kit, which allowed strand-specific RNA-seq. First, poly(A) selection using magnetic beads was performed to focus sequencing on polyadenylated transcripts. After fragmentation, cDNA synthesis was performed, and the resulting fragments were used for dA-tailing and were ligated to the TruSeq indexed adapters (for the TruSeq kit) or RNA Index Anchors (for the mRNA Ligation kit). PCR amplification was performed to generate the final indexed cDNA libraries (with 13 cycles). Individual library quantification and quality assessment was performed using a Qubit fluorometric assay (Invitrogen) with a dsDNA High-Sensitivity Assay kit and LabChip GX Touch using a High-Sensitivity DNA chip (PerkinElmer). Libraries were then pooled at equimolar concentrations and quantified by qPCR using a KAPA library quantification kit (Roche). Sequencing was performed on a NovaSeq 6000 instrument from Illumina using paired-end 2 × 100 bp sequencing to obtain around 30 million clusters (60 million raw paired-end reads) per sample.

### RNA-seq data analysis

Library read repartition (for example, for potential ribosomal contamination), inner distance size estimation, gene body coverage and strand specificity were performed using FastQC, Picard-Tools, Samtools and RseQC. Reads were mapped using STAR^61^ on the mm39 genome assembly.

Gene expression was estimated as described previously^62^ using Mouse FAST DB v2021_2 annotations. Only genes expressed in at least one of the two compared conditions were analyzed further. Genes were considered expressed if their fragments per kilobase per million mapped reads (FPKM) value was greater than the FPKM of 98% of the intergenic regions (background). Analysis at the gene level was performed using DESeq2 (ref. ^63^). Genes were considered differentially expressed if the fold change value was ≥1.5 and the *P* value was ≤0.05. Pathway analyses and transcription factor network analyses were performed using WebGestalt 0.4.4 (ref. ^64^), merging results from upregulated and downregulated genes only as well as all regulated genes. Pathways and networks were considered significant with a false discovery rate of ≤0.05. Graphics (heat map, MDS, scatter and volcano plots) were generated using R v4.2.1 with the help of pheatmap, ggplot2 and EnhancedVolcano^65^ packages, respectively. Heat maps were created using the *z* score of EdgeR-normalized counts.

### Analysis of microarray data

GSE49358 microarray data^39^ were downloaded from Gene Expression Omnibus (GEO) and annotated using the ‘mogene10stprobeset.db’ R package (v. 2.7). cPLA signature genes were displayed as heat maps using tidyverse (v. 1.3.0), scales (v. 1.1.1) and readxl (v. 1.3.1).

### In vivo analysis of DC subsets

For the in vivo analysis of DC subsets, both male and female wild-type and knockout littermates at the age of 8–12 weeks (*n* = 6 to 9 mice per experiment) were used. To collect cells from the skin, a section of skin (1 cm^2^) was cut and transferred to a 1-ml Eppendorf tube containing 0.25 mg ml^–1^ liberase (Sigma, 5401020001) and 0.5 mg ml^–1^ DNase (Sigma, 10104159001) in 1 ml of RPMI (Sigma). The skin was cut using scissors and incubated for 2 h at 37 °C. To collect cells from lymph nodes, inguinal lymph nodes were removed and transferred to an Eppendorf tube containing 500 µl of RPMI. DNase and collagenase were added at 0.5 mg ml^–1^ and 1 mg ml^–1^, respectively. The lymph nodes were further cut with scissors and incubated for 20 min at 37 °C. Cells were resuspended in PBS supplemented with 0.5% bovine serum albumin and 2 mM EDTA at 4 °C, filtered and stained following the same protocol as described earlier. The following antibodies were used: anti-CCR7 (Biolegend, 120114, clone 4B12; 1:50), anti-CD11c (eBioscience, 25-0114-81, clone N418; 1:800), anti-CD326 (Biolegend, 118217, clone G8.8; 1:800), anti-CD86 (BD Pharmingen, 553692, clone GL-1; 1:800), anti-CD11b (Biolegend, 101237, clone M1/70; 1:500), anti-MHC class II (eBioscience, 56-5321-80, clone I-A/I-E-M5/114.15.2; 1:250), anti-CD45 (eBioscience, 61-0451-82, clone 30-F11; 1:100), anti-CD8a (BD Bioscience, 553035, clone 53-6.7; 1:100) and anti-CD103 (eBioscience, 46-1031-80, clone 2E7; 1:100). Counting beads were used to normalize the number of cells to the number of beads. Flow cytometry was performed on an LSRII flow cytometer (BD Biosciences), and data were analyzed using FlowJo software version 10. Percentage values were plotted with GraphPad Prism version 8.

### Calculation of EOP_NE_

To calculate the EOP, we made a mask on the nucleus, then from the RIO function in ImageJ, we obtained the values of nuclear ‘area’ and ‘perimeter’ (*P*). We then calculated *R*_0_, which is the radius of a circle defined by the area of the nucleus. The ratio between *P* − 2π*R*_0_ and 2π*R*_0_ was calculated as the EOP_NE_. EOP values close to 1 indicate a highly folded (and thus less tensed) envelope, whereas EOP values close to 0 indicate a smooth surface with fewer folds (and thus a more tensed envelope).

### Calculation of EFC

Based on the work by Tamashunas et al.^66^, we fit contour with sum of successive Fourier ellipses (total number of ellipses for good fit of 25). We first made a mask of all the nucleus that needed to be analyzed and then automatically compared the relative contributions of the first-order ellipse to those of the later-order ellipses. The larger the EFC ratio, the more regular/smooth the shape. The EFC ratio is the sum of major and minor axes of the first ellipse divided by the sum of the major and minor axes of all others

### Multiphoton imaging of immune cells in mouse ear skin ex vivo

CD11c–EYFP mice were injected intravenously with 20 µl of CD31 (Thermo Fisher, 5278509) 5 min before being killed. Mouse ears were retrieved by cutting them with scissors and placing them in RPMI at 37 °C. Hair was removed with forceps. Ears were mechanically opened in two sheets with two forceps and were maintained in RPMI. The thick part of the posterior sheet was cut and prepared for imaging by piercing a piece of tape with several holes using a 29-G needle. Using a tissue, the epidermis of the posterior ear sheet was dried and placed on the pierced tape. The sheet was flattened as much as possible without damaging the dermis and placed at the bottom of a fluorodish, and 1 ml of preheated imaging medium was added. The tissue was imaged with a Biphoton microscope (×20; CD11c (orange), CD31 (blue) and fibrillar collagen (gray)).

### Calculation of the minimum and maximum diameter of DCs in ear skin

We selected several cells from the field of view and duplicated their corresponding movies. The cell masks were created using the thresholding function in ImageJ. Subsequently, we duplicated the movie with the mask and applied the ‘skeletonize’ function in ImageJ. This function tags all the pixels/voxels in a skeleton image and then counts all junctions, triple and quadruple points and branches. It automatically generated a region of interest with all the different frames of the cells. For the other mask’s movie, we applied the ‘distance map’ function, which generates a Euclidean distance map. Each pixel in the image was replaced with a gray value equal to the pixel’s distance from the nearest background pixel. The region of interest with the different frames was measured on the distance map of each frame for one cell. Measurements of the average, minimum and maximum lengths of each frame were calculated. We then calculated the average minimum (or maximum) diameter for one cell over several frames.

### Calculation of the time spent with a minimum cell diameter

For each cell, we duplicated the corresponding movie to track the cell over a minimum time scale of 40 min. We measured and recorded the minimum diameter of the cell at each time point throughout the entire duration of the movie. Subsequently, we calculated and plotted the time spent at a minimum diameter of 2–4 µm or at higher than 4 µm.

### FLIM measurements for nuclear envelope tension

HeLa Kyoto EMBO cells were cultured in DMEM supplemented with 10% FBS and 1% penicillin–streptomycin. ER Flipper-TR probe from Spirochrome (SC021) was added 30 minutes before the start of the experiment. For the FLIM experiments involving CK666, cells were plated in Fluorobright (Gibco) supplemented with 10% FBS, 1% penicillin–streptomycin, 1× GlutaMAX (Gibco) and 25 mM HEPES for 30 min and incubated with 30 µM CK666 and 1 µM ER Flipper-TR for 30 min.

### Statistics and data handling

Analyses were performed using GraphPad Prism 8 software. No statistical methods were used to predetermine sample sizes, but our sample sizes are similar to those reported in previous publications^21^. Data distribution was assumed to be normal (unless stated otherwise), but this was not formally tested. Mice were chosen randomly for the experiments. Experimental conditions were organized randomly. Data collection and analysis were not performed blind to the conditions of the experiments. No data points nor animals were excluded from the analysis.

### Reporting summary

Further information on research design is available in the Nature Portfolio Reporting Summary linked to this article.

## Online content

Any methods, additional references, Nature Portfolio reporting summaries, source data, extended data, supplementary information, acknowledgements, peer review information; details of author contributions and competing interests; and statements of data and code availability are available at 10.1038/s41590-024-01856-3.

### Supplementary information

Reporting Summary

Peer Review File

Supplementary Video 1 Example of a CD11c–YFP DC in yellow migrating in the ear dermis observed by second-harmonic generation, with collagen fibers in gray and lymphatics in blue. In a white box is a zoom in on one cell that constantly undergoes shape changes while migrating; time interval, 2 min; scale bar, 100 µm. Movie created with ImageJ.

Supplementary Video 2 Bone marrow-derived DCs from CCR7–GFP mice under confinement at heights of 4, 3 and 2 µm. Top, images of cells in transmission light (in gray) and GFP signal (in green). Bottom, GFP signal using false colors (Physics LUT). The warmer the color, the greater the intensity of GFP signal. The transmission images were corrected for bleach (with the function bleach correction in ImageJ), and GFP signal was smoothed using the Median filter. Time is shown in minutes (using the time stamper function). Movie created with ImageJ.

Supplementary Video 3 Example of NLS–GFP expression in bone marrow-derived DCs under confinement at a height of 2 µm. The movie shows examples from two different positions of the same condition displaying rupture (when the GFP signal is diffused) and repair (when the signal is concentrated in the nucleus) events. The stacks were merged using the stack-merge function in ImageJ. The movie is a maximum *z* projection of the GFP signal. Time is shown in minutes (using the time stamper function). Movie created with ImageJ.

Supplementary Video 4 Example 1 of immature DCs expressing LifeAct–GFP in gray and stained with NucBlue to visualize the nucleus (DNA) in red. Example 2 of immature DCs expressing LifeAct–GFP in green directly under confinement. Cells were stained with NucBlue to visualize the nucleus (DNA) in red. Movies are in *z* stack (step size of 0.33 µm). Top, *xy* images. Bottom, *xz* images. These data demonstrate the accumulation of an ARP2/3-dependent actin cloud in the perinuclear area when the cells were under confinement at a height of 3 µm. Movie created with ImageJ.

Supplementary Video 5 A movie of live WASp^WT^ (left) or WASp^KO^ (right) DCs under confinement at a height of 3 µm. These data demonstrate that both cell types are alive; however, WASp^KO^ cells stay round and do not move as much as the WASp^WT^ cells; timelapse of 2 min. Time is shown in minutes (using the time stamper function). Movie created with ImageJ.

### Source data

Source Data Fig. 1 Statistical source data.

Source Data Fig. 2 Statistical source data.

Source Data Fig. 3 Statistical source data.

Source Data Fig. 4 Statistical source data.

Source Data Fig. 5 Statistical source data.

Source Data Fig. 6 Statistical source data.

Source Data Fig. 7 Statistical source data.

**Extended Data Fig. 7:**
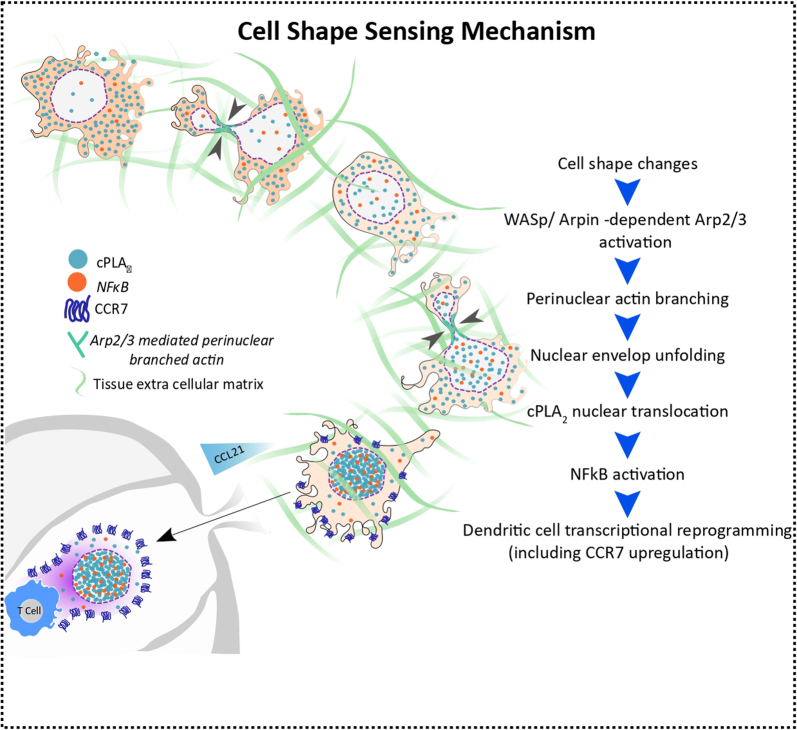
A model figure. A model figure representing cell shape sensing pathway in patrolling dendritic cells, created with Inkscape. Source data

Source Data Extended Data Figs. 1–7 Statistical source data for all Extended Data figures.

## Data Availability

RNA-seq data have been deposited in the GEO under the accession code GSE207653. Raw data quantification used to generate figures are available on FigShare at 10.6084/m9.figshare.25236715 (ref. ^67^). GSE49358 microarray data were accessed at https://www.ncbi.nlm.nih.gov/geo/query/acc.cgi?acc=GSE49358. The confinement protocol is deposited on Protocol Exchange at 10.21203/rs.3.pex-2616/v1 (ref. ^68^). Source data are provided with this paper.

## Code availability

Scripts of the macros used to analyze microscopy images are available at https://github.com/Zalraies/Alraies-at-al-2024n.
