# Supplementary material for: Cell shape sensing licenses dendritic cells for homeostatic migration to lymph nodes
Source: Nat Immunol. 2024 Jun 4;25(7):1193–206. doi: 10.1038/s41590-024-01856-3 (PMC11224020; doi:10.1038/s41590-024-01856-3)
Supplement: Supplementary file 1 — Reporting Summary [file 41590_2024_1856_MOESM1_ESM.pdf]

nature portfolio

Corresponding author(s): Ana Maria Lennon Duménil
Matthieu Piel

Last updated by author(s): 16/04/2024

Reporting Summary

Nature Portfolio wishes to improve the reproducibility of the work that we publish. This form provides structure for consistency and transparency in reporting. For further information on Nature Portfolio policies, see our Editorial Policies and the Editorial Policy Checklist.

Statistics

For all statistical analyses, confirm that the following items are present in the figure legend, table legend, main text, or Methods section.

n/a	Confirmed	
☐	☒	The exact sample size (*n*) for each experimental group/condition, given as a discrete number and unit of measurement
☐	☒	A statement on whether measurements were taken from distinct samples or whether the same sample was measured repeatedly
☐	☒	The statistical test(s) used AND whether they are one- or two-sided<br>*Only common tests should be described solely by name; describe more complex techniques in the Methods section.*
☒	☐	A description of all covariates tested
☐	☒	A description of any assumptions or corrections, such as tests of normality and adjustment for multiple comparisons
☐	☒	A full description of the statistical parameters including central tendency (e.g. means) or other basic estimates (e.g. regression coefficient) AND variation (e.g. standard deviation) or associated estimates of uncertainty (e.g. confidence intervals)
☐	☒	For null hypothesis testing, the test statistic (e.g. F, t, r) with confidence intervals, effect sizes, degrees of freedom and P value noted<br>*Give P values as exact values whenever suitable.*
☒	☐	For Bayesian analysis, information on the choice of priors and Markov chain Monte Carlo settings
☒	☐	For hierarchical and complex designs, identification of the appropriate level for tests and full reporting of outcomes
☒	☐	Estimates of effect sizes (e.g. Cohen's *d*, Pearson's *r*), indicating how they were calculated

Our web collection on statistics for biologists contains articles on many of the points above.

Software and code

Policy information about availability of computer code

Data collection	All microscopes were controlled by Meta Morphe software for data collection
Data analysis	Image analysis was performed using ImagJ software (version ImageJ2)., For image analysis custom made codes from ImageJ were used and they are available on Guithub (https://github.com/Zalraies/Alraies-at-al-2024).For FACS: data were analyzed using FlowJo software (version 10). All statistical analysis was done by Graph pad prism (version 8). For RNA seq data: Gene expression was estimated as described previously [PMID: 34495298] using Mouse FAST DB v2021_2 annotations, Pathway analyses and transcription factor network analysis were performed using WebGestalt 0.4.4 (92)[PMID: 31114916], Microarray data were annotated using the 'mogene10stprobeset.db' R package (v. 2.7). cPLA2 signature genes were displayed as heatmaps using tidyverse (v. 1.3.0), scales (v. 1.1.1), readxl (v. 1.3.1). Heat map, MDS, scatter and volcano plots were generated using R v4.2.1 with the help of pheatmap, ggplot2, and EnhancedVolcano [PMID:27207943] packages respectively. The heat maps were created using the Z-score of EdgeR normalized counts.

For manuscripts utilizing custom algorithms or software that are central to the research but not yet described in published literature, software must be made available to editors and reviewers. We strongly encourage code deposition in a community repository (e.g. GitHub). See the Nature Portfolio guidelines for submitting code & software for further information.

Data

Policy information about availability of data

All manuscripts must include a data availability statement. This statement should provide the following information, where applicable:

- Accession codes, unique identifiers, or web links for publicly available datasets
- A description of any restrictions on data availability
- For clinical datasets or third party data, please ensure that the statement adheres to our policy

Raw RNA-seq data are available at GEO under the number: GSE207653: https://www.ncbi.nlm.nih.gov/geo/query/acc.cgi
Raw data quantification used to generate the figures are available on FigShare: DOI: 10.6084/m9.figshare.25236715
The GSE49358 microarray data (Tamoutounour et al., 2013) were downloaded from GE: https://www.ncbi.nlm.nih.gov/geo/query/acc.cgi?acc=GSE49358
Confinement protocol is deposited on protocol exchange, DIO: https://doi.org/10.21203/rs.3.pex-2616/v1

Research involving human participants, their data, or biological material

Policy information about studies with human participants or human data. See also policy information about sex, gender (identity/presentation), and sexual orientation and race, ethnicity and racism.

Reporting on sex and gender	NA
Reporting on race, ethnicity, or other socially relevant groupings	NA
Population characteristics	NA
Recruitment	NA
Ethics oversight	NA

Note that full information on the approval of the study protocol must also be provided in the manuscript.

Field-specific reporting

Please select the one below that is the best fit for your research. If you are not sure, read the appropriate sections before making your selection.

☒ Life sciences ☐ Behavioural & social sciences ☐ Ecological, evolutionary & environmental sciences

For a reference copy of the document with all sections, see nature.com/documents/nr-reporting-summary-flat.pdf

Life sciences study design

All studies must disclose on these points even when the disclosure is negative.

Sample size	No statistical methods were used to pre-determine sample sizes but our sample sizes are similar to those reported in previous publications (Lomakin et al., Science 2020).
Data exclusions	No data were excluded
Replication	Statistical analysis was carried out using Prism 8 (GraphPad Software). Statistical analysis was conducted on data from three or more biologically independent experimental replicates. Data distribution was assumed to be normal (unless stated otherwise), but this was not formally tested. Statistical significance was calculated between two groups by Mann-Whitney test. Ordinary Mann-Whitney with multiple comparison was used to calculate statistical significance between multiple groups. Analyses were performed using GraphPad Prism 8 software.
Randomization	Experimental conditions were organized randomly, mice were chosen randomly for the experiments.
Blinding	Data collection and analysis were not performed blind to the conditions of the experiments

Behavioural & social sciences study design

All studies must disclose on these points even when the disclosure is negative.

Study description	*Briefly describe the study type including whether data are quantitative, qualitative, or mixed-methods (e.g. qualitative cross-sectional,*

Study description	*quantitative experimental, mixed-methods case study).*
Research sample	*State the research sample (e.g. Harvard university undergraduates, villagers in rural India) and provide relevant demographic information (e.g. age, sex) and indicate whether the sample is representative. Provide a rationale for the study sample chosen. For studies involving existing datasets, please describe the dataset and source.*
Sampling strategy	*Describe the sampling procedure (e.g. random, snowball, stratified, convenience). Describe the statistical methods that were used to predetermine sample size OR if no sample-size calculation was performed, describe how sample sizes were chosen and provide a rationale for why these sample sizes are sufficient. For qualitative data, please indicate whether data saturation was considered, and what criteria were used to decide that no further sampling was needed.*
Data collection	*Provide details about the data collection procedure, including the instruments or devices used to record the data (e.g. pen and paper, computer, eye tracker, video or audio equipment) whether anyone was present besides the participant(s) and the researcher, and whether the researcher was blind to experimental condition and/or the study hypothesis during data collection.*
Timing	*Indicate the start and stop dates of data collection. If there is a gap between collection periods, state the dates for each sample cohort.*
Data exclusions	*If no data were excluded from the analyses, state so OR if data were excluded, provide the exact number of exclusions and the rationale behind them, indicating whether exclusion criteria were pre-established.*
Non-participation	*State how many participants dropped out/declined participation and the reason(s) given OR provide response rate OR state that no participants dropped out/declined participation.*
Randomization	*If participants were not allocated into experimental groups, state so OR describe how participants were allocated to groups, and if allocation was not random, describe how covariates were controlled.*

Ecological, evolutionary & environmental sciences study design

All studies must disclose on these points even when the disclosure is negative.

Study description	*Briefly describe the study. For quantitative data include treatment factors and interactions, design structure (e.g. factorial, nested, hierarchical), nature and number of experimental units and replicates.*
Research sample	*Describe the research sample (e.g. a group of tagged Passer domesticus, all Stenocereus thurberi within Organ Pipe Cactus National Monument), and provide a rationale for the sample choice. When relevant, describe the organism taxa, source, sex, age range and any manipulations. State what population the sample is meant to represent when applicable. For studies involving existing datasets, describe the data and its source.*
Sampling strategy	*Note the sampling procedure. Describe the statistical methods that were used to predetermine sample size OR if no sample-size calculation was performed, describe how sample sizes were chosen and provide a rationale for why these sample sizes are sufficient.*
Data collection	*Describe the data collection procedure, including who recorded the data and how.*
Timing and spatial scale	*Indicate the start and stop dates of data collection, noting the frequency and periodicity of sampling and providing a rationale for these choices. If there is a gap between collection periods, state the dates for each sample cohort. Specify the spatial scale from which the data are taken*
Data exclusions	*If no data were excluded from the analyses, state so OR if data were excluded, describe the exclusions and the rationale behind them, indicating whether exclusion criteria were pre-established.*
Reproducibility	*Describe the measures taken to verify the reproducibility of experimental findings. For each experiment, note whether any attempts to repeat the experiment failed OR state that all attempts to repeat the experiment were successful.*
Randomization	*Describe how samples/organisms/participants were allocated into groups. If allocation was not random, describe how covariates were controlled. If this is not relevant to your study, explain why.*
Blinding	*Describe the extent of blinding used during data acquisition and analysis. If blinding was not possible, describe why OR explain why blinding was not relevant to your study.*

Did the study involve field work? ☐ Yes ☒ No

Field work, collection and transport

Field conditions	*Describe the study conditions for field work, providing relevant parameters (e.g. temperature, rainfall).*
Location	*State the location of the sampling or experiment, providing relevant parameters (e.g. latitude and longitude, elevation, water depth).*
Access & import/export	*Describe the efforts you have made to access habitats and to collect and import/export your samples in a responsible manner and in*

Access & import/export	*compliance with local, national and international laws, noting any permits that were obtained (give the name of the issuing authority, the date of issue, and any identifying information).*
Disturbance	*Describe any disturbance caused by the study and how it was minimized.*

Reporting for specific materials, systems and methods

We require information from authors about some types of materials, experimental systems and methods used in many studies. Here, indicate whether each material, system or method listed is relevant to your study. If you are not sure if a list item applies to your research, read the appropriate section before selecting a response.

Materials & experimental systems

n/a	Involved in the study	
☐	☒	Antibodies
☐	☒	Eukaryotic cell lines
☒	☐	Palaeontology and archaeology
☐	☒	Animals and other organisms
☒	☐	Clinical data
☒	☐	Dual use research of concern
☒	☐	Plants

Methods

n/a	Involved in the study	
☒	☐	ChIP-seq
☐	☒	Flow cytometry
☒	☐	MRI-based neuroimaging

Antibodies

Antibodies used	For DC in LN FACS analysis: Anti-CCR7 (Biolegend #120114, clone 4B12, dilution: 1/50), anti-CD11c (eBioscience #25-0114-81, clone: N418, dilution: 1/800), anti-CD326 (Biolegend #118217, clone: G8.8, dilution: 1/800), anti-CD86 (BD pharmingen #553692, clone: GL1, dilution: 1/800), anti-CD11b (Biolegend #101237, clone: M1/70, dilution : 1/500), anti-MHCII (eBioscience #56-5321-80, clone: I-A/I-E - M5/114.15.2, dilution: 1/250), anti-CD45 (eBioscience #61-0451-82, clone: 30-F11, dilution: 1/100), anti-CD8a (BD bioscience #553035, clone: 53-6.7, dilution: 1/100), anti-CD103 (eBioscience #46-1031-80, clone: 2E7, dilution: 1/100).<br>For T cell FACS analysis: anti-CD4 (BD # 553051, clone RM4-5, dilution: 1/100), anti-CD69 (eBioscience # 48-0691-82, clone H1.2F3, dilution: 1/300), anti-TCR (BD # 553190, clone MR9-4, dilution: 1/300).<br>For DC FACS analysis: anti-CD80 (BD # 553769, clone 16-10A1, dilution: 1/100), anti-MHCII (Ozym # BLE107622, clone M5/114.15.2, dilution 1/400), anti-CD86 (Biolegend # 105037, clone GL-1, dilution: 1/200), anti-CD11c (BD # 550261, clone HL3, dilution: 1/200), Fc antibody (BD #553142, dilution 1/1000).<br>For IF: anti-CCR7 (abcam #ab32527, dilution : 1/100), anti-cPLA2 (cell signalling (Ozyme) #2832, dilution 1/100), anti-NF-κB P65 (cell signalling #mAB 8242, dilution 1/200), anti-Lap2 (BD Bioscience # 611000, dilution 1/200), Alexa Fluor-coupled Phalloidin (Invitrogen, dilution 1/300).<br>For secondary antibodies the following were used: Alexa 647 goat anti rabbit (Invitrogen, A21244, 1/300) and Alexa 488 goat anti mouse Fab2 (Invitrogen, A11017, 1/300).
Validation	All antibodies were validated on KO cells

Eukaryotic cell lines

Policy information about cell lines and Sex and Gender in Research

Cell line source(s)	HeLa EMBL (Kyoto) were from Valerie Doyen, J588 cell lines were from S. Amigorena (Institut Curie). Both cell lines are originally commercially available on American Type Culture Collection ATCC.
Authentication	No authentication is done
Mycoplasma contamination	All cell lines tested negative for mycoplasma contamination
Commonly misidentified lines (See ICLAC register)	No misidentified cell lines were used in the study

Palaeontology and Archaeology

Specimen provenance	*Provide provenance information for specimens and describe permits that were obtained for the work (including the name of the issuing authority, the date of issue, and any identifying information). Permits should encompass collection and, where applicable, export.*
Specimen deposition	*Indicate where the specimens have been deposited to permit free access by other researchers.*
Dating methods	*If new dates are provided, describe how they were obtained (e.g. collection, storage, sample pretreatment and measurement), where*

Dating methods	*they were obtained (i.e. lab name), the calibration program and the protocol for quality assurance OR state that no new dates are provided.*

☐ Tick this box to confirm that the raw and calibrated dates are available in the paper or in Supplementary Information.

Ethics oversight	*Identify the organization(s) that approved or provided guidance on the study protocol, OR state that no ethical approval or guidance was required and explain why not.*

Note that full information on the approval of the study protocol must also be provided in the manuscript.

Animals and other research organisms

Policy information about studies involving animals; ARRIVE guidelines recommended for reporting animal research, and Sex and Gender in Research

Laboratory animals	For animal care, we strictly followed the European and French National Regulation for the Protection of Vertebrate Animals used for Experimental and other Scientific Purposes (Directive 2010/63; French Decree 2013-118, Authorization DAP number 43530-2023051216135493 v2 given by National Authority). Experiments were performed on 8 to 14 weeks-old male or female mice. Mice were maintained under specific pathogen-free conditions at the animal facility of Institut Curie, in accordance with institutional guidelines, housed in a 12h light:12h dark environment with free access to water (osmotic water) and food (MUCEDOLA - 4RF25SV Aliment Pellets 8*16mm irradiated 2,5 Mrad, and DietGel® Recovery, 2 oz / DietGel® Boost, 2 oz) . Littermates or age-mated mice were used as controls for all experiments involving knockout animals; breeder mice were previously backcrossed to C57BL6 for 7generations.C57BL6/J mice were obtained from Charles River, catalog #000664. CCR7-GFP mice were obtained from Jackson laboratory (stock# 027913), bred in our animal facility, original paper: Nakano H et al., 2013. CD11c-Cre mice: bred in our animal facility (Caton et al., 2007). Arpin and cPLA2 conditional knockout mice were generated by CIPHE (Centre d'Immunophénomique) Marseille, France. Both mice were generated using CRISPR cas-9 technique to generate cPLA2 and Arpin Flox mice that were crossed in our animal facility with Cd11c Cre mice. Ikkb knockout mice were from Toby Lawernce laboratory described in Baratin et al., 2015. WASp KO mice on a C57BL/6 (CD45.2) genetic background were from Federica Benvenuti lab mice. Experiments were performed using homozygous WASp –/– as KO. Lmna fl/fl Vav1-Cre+/– (lamin A/C CK) mice were obtained from the lab of Nicolas Manel. YFP CD11c mice: From the lab of S. Amigorena (original paper: Lindquist et al, 2004).
Wild animals	The study didn't involve wild animals
Reporting on sex	Males and females were used
Field-collected samples	No field collected samples were used in this study
Ethics oversight	For animal care, we strictly followed the European and French National Regulation for the Protection of Vertebrate Animals used for Experimental and other Scientific Purposes (Directive 2010/63; French Decree 2013-118, Authorization DAP number 43530-2023051216135493 v2 given by National Authority).

Note that full information on the approval of the study protocol must also be provided in the manuscript.

Clinical data

Policy information about clinical studies

All manuscripts should comply with the ICMJE guidelines for publication of clinical research and a completed CONSORT checklist must be included with all submissions.

Clinical trial registration	*Provide the trial registration number from ClinicalTrials.gov or an equivalent agency.*
Study protocol	*Note where the full trial protocol can be accessed OR if not available, explain why.*
Data collection	*Describe the settings and locales of data collection, noting the time periods of recruitment and data collection.*
Outcomes	*Describe how you pre-defined primary and secondary outcome measures and how you assessed these measures.*

Dual use research of concern

Policy information about dual use research of concern

Hazards

Could the accidental, deliberate or reckless misuse of agents or technologies generated in the work, or the application of information presented in the manuscript, pose a threat to:

No	Yes	
☒	☐	Public health
☒	☐	National security
☒	☐	Crops and/or livestock
☒	☐	Ecosystems
☒	☐	Any other significant area

Experiments of concern

Does the work involve any of these experiments of concern:

No	Yes	
☒	☐	Demonstrate how to render a vaccine ineffective
☒	☐	Confer resistance to therapeutically useful antibiotics or antiviral agents
☒	☐	Enhance the virulence of a pathogen or render a nonpathogen virulent
☒	☐	Increase transmissibility of a pathogen
☒	☐	Alter the host range of a pathogen
☒	☐	Enable evasion of diagnostic/detection modalities
☒	☐	Enable the weaponization of a biological agent or toxin
☒	☐	Any other potentially harmful combination of experiments and agents

Plants

Seed stocks	*Report on the source of all seed stocks or other plant material used. If applicable, state the seed stock centre and catalogue number. If plant specimens were collected from the field, describe the collection location, date and sampling procedures.*
Novel plant genotypes	*Describe the methods by which all novel plant genotypes were produced. This includes those generated by transgenic approaches, gene editing, chemical/radiation-based mutagenesis and hybridization. For transgenic lines, describe the transformation method, the number of independent lines analyzed and the generation upon which experiments were performed. For gene-edited lines, describe the editor used, the endogenous sequence targeted for editing, the targeting guide RNA sequence (if applicable) and how the editor was applied.*
Authentication	*Describe any authentication procedures for each seed stock used or novel genotype generated. Describe any experiments used to assess the effect of a mutation and, where applicable, how potential secondary effects (e.g. second site T-DNA insertions, mosiacism, off-target gene editing) were examined.*

Flow Cytometry

Plots

Confirm that:

☒ The axis labels state the marker and fluorochrome used (e.g. CD4-FITC).

☒ The axis scales are clearly visible. Include numbers along axes only for bottom left plot of group (a 'group' is an analysis of identical markers).

☒ All plots are contour plots with outliers or pseudocolor plots.

☒ A numerical value for number of cells or percentage (with statistics) is provided.

Methodology

Sample preparation	cells were recovered directly by gently washing with medium.Cells were re-suspended in the buffer (PBS BSA 1% EDTA 2mM). After blocking with Fc antibody (BD #553142) and live/dead staining kit (Thermo # L34966) for 15 min, cells were stained with the desired antibodies for 20 min Cells were then washed three times and re-suspended in the staining buffer
Instrument	Flow cytometry was performed on LSRII (BD)
Software	FlowJo software version 10
Cell population abundance	cells of interest were highly abudant in the culture and were defined by a gating strategy and checked for vaiability using live dead staining whenever applicable
Gating strategy	Gating strategy to quantify DCs in skin-draining lymph nodes and skin of mice at steady-state: after gating on live cells, immune cells were identified as CD45high; CD11c and MHCII were then used to differentiate lymph node-resident DCs

(MHCII low, CD11chigh) from migratory DCs (MHCIIhigh, CD11chigh). Among migratory cDCs, mDC1s were identified as CD11bhigh, CD103low and mDC2s as CD11bhigh, EPCAMlow.

☒ Tick this box to confirm that a figure exemplifying the gating strategy is provided in the Supplementary Information.
